# Causal associations between gut microbiota, metabolites and asthma: a two-sample Mendelian randomization study

**DOI:** 10.1186/s12890-024-02898-x

**Published:** 2024-02-07

**Authors:** Jingli Li, Chunyi Zhang, Jixian Tang, Meng He, Chunxiao He, Guimei Pu, Lingjing Liu, Jian Sun

**Affiliations:** 1 Department of Pulmonary and Critical Care Medicine, Shaoxing People’s Hospital, Shaoxing, 312000 Zhejiang China; 2 Department of Pulmonary and Critical Care Medicine, The First Affiliated Hospital of Wenzhou Medical University, Wenzhou, 325000 Zhejiang China

**Keywords:** Gut microbiota, Mendelian randomization study, Asthma, Gut metabolites

## Abstract

**Background:**

While several traditional observational studies have suggested associations between gut microbiota and asthma, these studies are limited by factors such as participant selection bias, confounders, and reverse causality. Therefore, the causal relationship between gut microbiota and asthma remains uncertain.

**Methods:**

We performed two-sample bi-directional Mendelian randomization (MR) analysis to investigate the potential causal relationships between gut microbiota and asthma as well as its phenotypes. We also conducted MR analysis to evaluate the causal effect of gut metabolites on asthma. Genetic variants for gut microbiota were obtained from the MiBioGen consortium, GWAS summary statistics for metabolites from the TwinsUK study and KORA study, and GWAS summary statistics for asthma from the FinnGen consortium. The causal associations between gut microbiota, gut metabolites and asthma were examined using inverse variance weighted, maximum likelihood, MR-Egger, weighted median, and weighted model and further validated by MR-Egger intercept test, Cochran’s Q test, and “leave-one-out” sensitivity analysis.

**Results:**

We identified nine gut microbes whose genetically predicted relative abundance causally impacted asthma risk. After FDR correction, significant causal relationships were observed for two of these microbes, namely the class *Bacilli* (OR = 0.84, 95%CI = 0.76–0.94, *p* = 1.98 × 10^−3^) and the order *Lactobacillales* (OR = 0.83, 95%CI = 0.74–0.94, *p* = 1.92 × 10^−3^). Additionally, in a reverse MR analysis, we observed a causal effect of genetically predicted asthma risk on the abundance of nine gut microbes, but these associations were no longer significant after FDR correction. No significant causal effect of gut metabolites was found on asthma.

**Conclusions:**

Our study provides insights into the development mechanism of microbiota-mediated asthma, as well as into the prevention and treatment of asthma through targeting specific gut microbiota.

**Supplementary Information:**

The online version contains supplementary material available at 10.1186/s12890-024-02898-x.

## Background

Asthma is a common chronic respiratory disease worldwide, typically starting in childhood and characterized by symptoms such as shortness of breath, chest tightness, wheezing, and coughing, which may vary in frequency and severity over time [1]. It is estimated that asthma-related symptoms impact a substantial global population, with the most recent Global Burden of Disease Study (2019) reporting an asthma prevalence of nearly 262 million individuals worldwide [2]. The condition may be particularly severe in some children with asthma, especially in low- and middle-income countries [3]. Cluster analysis has identified distinct asthma phenotypes among patients, which are influenced by various factors such as age at asthma onset, sex, body mass index (BMI), and inflammatory profiles [4]. The etiology of asthma is complex and likely involves a variety of genetic, environmental, infectious, and nutritional factors [5]. Some environmental factors that may contribute to the onset and exacerbation of asthma include allergens, viral infections, tobacco smoke exposure, and air pollution [6]. The development of asthma may also be related to individual susceptibility. Despite significant progress in understanding and managing asthma, it remains a major public health problem with substantial morbidity, mortality, and economic burden [7].

The gut microbiota plays a critical role in regulating human health through various mechanisms such as metabolic and immune regulation [8]. Environmental factors, including antibiotic use and birth mode, have been shown to impact the gut microbiota composition and increase the vulnerability to immune-related disease [9, 10]. Dysbiosis of the gut microbiota, characterized by an altered microbial community composition and imbalance, has been correlated to various diseases, such as obesity, hypertension, diabetes, and cancer [11–13]. Recently, several cross-sectional studies have shown the association between gut microbiota and asthma. For example, Demirci et al. found that *Akkermansia muciniphila* and *Faecalibacterium prausnitzii* were decreased in the asthma children group compared to the healthy children group [14]. A Danish prospective cohort study on asthma indicates that a higher abundance of *Veillonella* and lower abundance of *Roseburia*, *Alistipes*, and *Flavonifractor* at age 1 year were associated with an increased risk of developing asthma by age 5 years [15]. While these traditional observational studies have suggested associations between gut microbiota and asthma, these studies are limited by factors such as participant selection bias, confounders, and reverse causality. Therefore, the causal relationship between gut microbiota and asthma remains uncertain. It is imperative to clarify a causal relationship to better understand the pathogenesis of asthma and guide microbiota-oriented interventions in clinical practice.

Mendelian randomization (MR) is a statistical method that infers the causal relationship between exposures and outcomes by using genetic variations as instrumental variables (IVs) [16]. MR integrates summary data from genome-wide association studies (GWAS), similar to natural randomized controlled trials. As the assignment of genotypes from parents to offspring is random, MR studies are less prone to confounders and reverse causality than traditional observational studies [17]. MR has emerged as a powerful tool for identifying causal relationships between risk factors and diseases and is widely used in epidemiological research to explore the potential causal associations between two traits [18].

Recently, MR analysis has been widely used to identify the causal associations between gut microbiota and the risk of many diseases, such as cardiovascular diseases, autoimmune diseases, and psychiatric disorders [19–21]. To our knowledge, no MR study has extensively examined the causal association between gut microbiota and asthma. Therefore, in this study, we conducted the two-sample bi-directional MR analysis to examine the potential causal relationships between gut microbiota and asthma as well as its phenotypes (i.e., obesity related asthma, non-allergic asthma, allergic asthma, and eosinophilic asthma). We also used the MR method to evaluate the causal effect of gut metabolites on asthma and its phenotypes.

## Methods

### Data sources

We obtained summary statistics for gut microbiota through the largest genome-wide meta-analysis conducted to date by the MiBioGen consortium [22]. This study encompassed an analysis of 16S rRNA gene sequencing profiles and genotyping data from 18,340 individuals across 24 population-based cohorts, with the aim of investigating the associations between host genetics and gut microbiome. Pool data for 9 phyla, 16 classes, 20 orders, 35 families, and 131 genera of gut microbiota were obtained [23]. Among the 211 gut microbiota taxa, the unknown gut microbiota taxa were excluded, and the 196 known taxa were eventually analyzed in the MR analysis [24, 25]. The composition of gut microbiota was characterized by sequencing three variable regions (V1-V2, V3-V4, and V4) of the 16S rRNA gene. To elucidate the genetic determinants influencing the relative abundance of microbial taxa, we performed Spearman’s correlation analysis. This method was selected for its non-parametric nature, which does not assume a normal distribution of the data, making it suitable for the skewed distributions often observed in microbial abundance data. In this analysis, we adjusted for potential confounders including age, gender, and technical covariates, such as batch effects and sample processing variations. Additionally, we accounted for population stratification by adjusting for principal genetic components. This adjustment is crucial to reduce false positives that may arise due to population structure rather than true genetic associations.

We utilized summary statistics obtained from a GWAS of blood metabolites in a cohort comprising 7824 individuals of European ancestry, derived from two distinct datasets (the British TwinsUK study, *n* = 6056; the German KORA F4 study, *n* = 1768) [26]. A total of 529 metabolites were measured in human plasma or serum samples, and a subset consisting of 486 metabolites was selected for genetic analysis in the GWAS after rigorous quality control procedures. The study has identified 145 metabolic loci with genome-wide significant associations, thus offering novel perspectives on the contribution of genetic variation to blood metabolic diversity. In selecting the thirteen microbe-derived metabolites for our analysis, including betaine, carnitine, choline, indolepropionate, glutamate, kynurenine, phenylalanine, serotonin, tryptophan, tyrosine, leucine, isoleucine, and valine, we were guided by emerging evidence suggesting their potential involvement in asthma pathogenesis. For instance, betaine and carnitine have been associated with alterations in the gut microbiota composition, which may influence asthma development [27, 28]. Similarly, tryptophan and its metabolites play a crucial role in immune regulation, which is pivotal in asthma’s etiology [29, 30]. The selection of these metabolites was based on their known or proposed roles in inflammatory processes, immune response modulation, and the gut-lung axis, all of which are critical in understanding asthma’s complex pathophysiology.

**Fig. 1:**
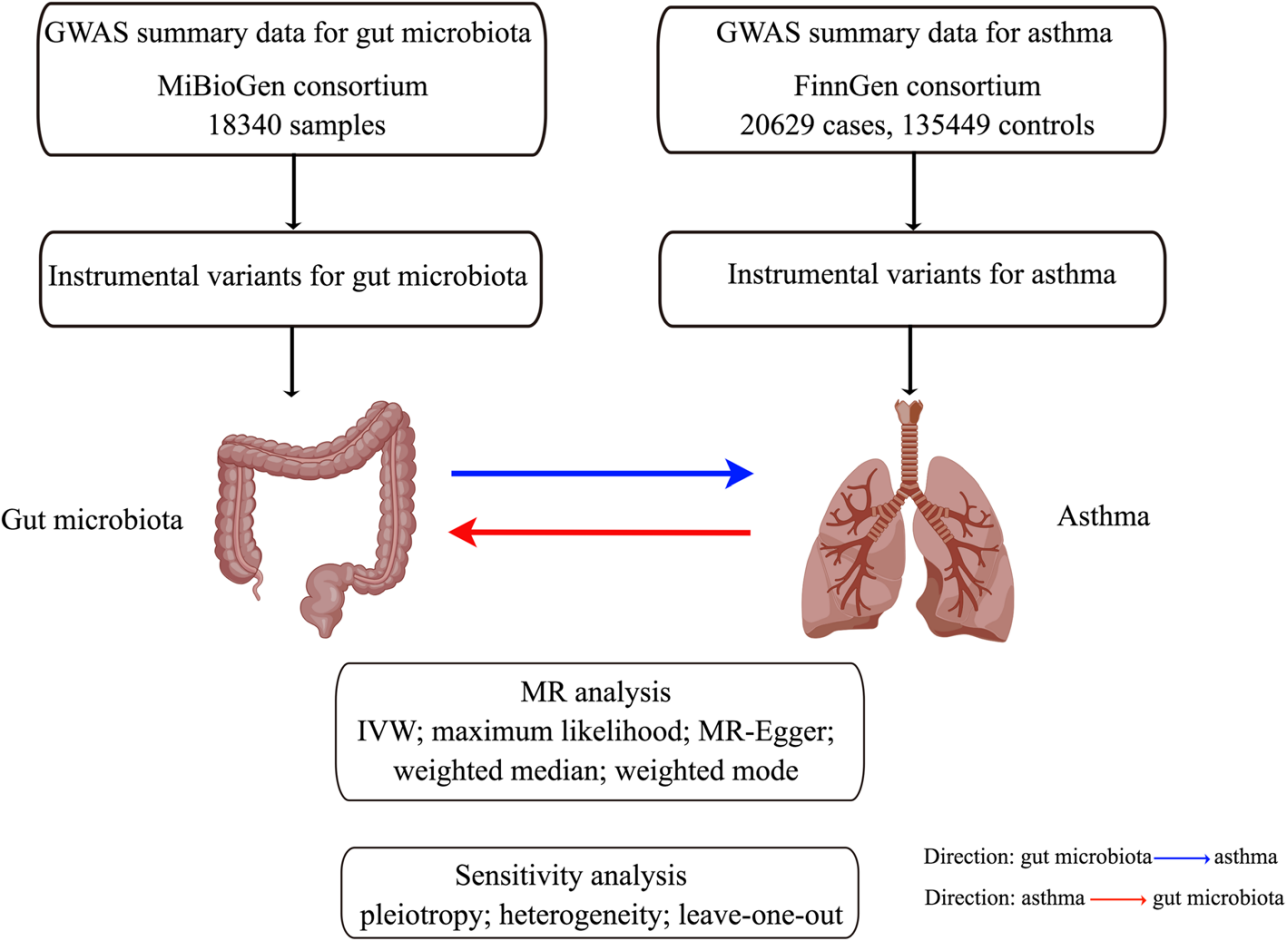
Flowchart of the two-sample bidirectional Mendelian randomization analysis of our study

GWAS summary statistics for asthma in this study were obtained from the FinnGen consortium [31]. The FinnGen project, initiated in 2017, involves a cohort of 500,000 individuals, with the primary aim of integrating genetic data with health-related information to advance human well-being through genetic research. This GWAS of asthma included 156,078 Finnish participants and consisted of 20,629 cases of (overall) asthma and 135,449 controls. Four asthma phenotypes, including obesity related asthma (*n* = 4142), non-allergic asthma (*n* = 3155), allergic asthma (*n* = 4859), and eosinophilic asthma (*n* = 1184) were further analyzed as secondary outcomes in our MR analysis. The detail of GWAS summary-level data is showed in Additional file 1: Table S1. The flowchart of this study is showed in Fig. 1.

### Instrumental variables selection

To ensure the robustness of data and the reliability of conclusions, we performed the following quality control steps in the selection of eligible IVs. Firstly, candidate IVs were identified by selecting single-nucleotide polymorphisms (SNPs) associated with gut bacterial taxa at the genome-wide significance level (*p* < 5 × 10^−8^). Due to the small number of loci identified for gut microbiota, we used the locus-wide significance (*p* < 1.0 × 10^−5^) threshold to obtain a more comprehensive result [32]. Similarly, we extracted SNPs at a threshold of p < 5 × 10^−5^ as IVs in the MR analysis of gut metabolites and asthma. Secondly, we ensured the independence of each SNP by setting the linkage disequilibrium (LD) threshold for clustering to be r^2^ < 0.001 and the size of the clumping distance to 10,000 kb, based on the European-based 1000 Genomes project reference panel [33]. We also harmonized the gut microbiota, metabolites, and asthma data for subsequent MR. We excluded SNPs with a minor allele frequency (MAF) less than 0.01. If SNPs associated to exposure were missing in the outcome GWAS, we selected proxy SNPs with an r^2^ > 0.80. Subsequently, we removed palindromic SNPs (with A/T or G/C alleles) to ensure that the allelic effects of SNPs on exposures were consistent with the allelic effects of SNPs on outcomes. Lastly, the PhenoScanner database (http://www.phenoscanner.medschl.cam.ac.uk/) was used to explore whether the identified SNPs were associated with the possible confounders for asthma [34].

### Statistical analysis

We conducted MR analyses to estimate the causal effect of the gut microbiota and gut metabolites on asthma and its phenotypes, using the Inverse-variance weighted (IVW) method as our main analysis approach [35]. When bacterial genera containing only one IV, we utilized the Wald ratio method. We also applied the maximum likelihood [36], MR-Egger regression [37], weighted median method [38], and weighted mode to further validate the robustness of MR analysis results when bacterial genera containing multiple IVs. If the IVW method yielded a significant result (*p* < 0.05), it was considered a positive result even if the other methods did not show significance, as long as the beta values of the other methods were in the same direction. To consider multiple-testing, we employed a modified version of the Benjamini and Hochberg false discovery rate (FDR) procedure, tailored to our data’s hierarchical and interdependent nature [39]. The FDR-corrected significance threshold at each taxonomic level was set as 0.05 divided by the effective number of independent tests at each taxonomic level: phylum *p* = 0.05/9 = 5.56 × 10^−3^, class *p* = 0.05/16 = 3.13 × 10^−3^, order *p* = 0.05/20 = 2.50 × 10^−3^, family *p* = 0.05/35 = 1.43 × 10^−3^, and genus *p* = 0.05/131 = 3.82 × 10^−4^. To identify potentially causal associations, we employed a significance threshold of *p* < 0.05, while also considering suggestive associations with FDR-corrected *p*-values greater than 0.05. We only included MR results with consistent effect estimates across all methods in further pleiotropy and heterogeneity testing. We used the MR-Egger regression and Mendelian Randomization Pleiotropy RESidual Sum and Outlier (MR-PRESSO) to identify the potential horizontal pleiotropy [37]. If the intercept of the MR-Egger had no statistical significance (*p* > 0.05), there was no evidence of the presence of horizontal pleiotropy. We performed the Cochrane’s Q statistic in the IVW test and MR- Egger regression to examine potential heterogeneity among the selected IVs [40]. If heterogeneity was the presence (*p* < 0.05), the random-effects IVW model was applied again to obtain a more unbiased and robust estimate. Additionally, we employed the leave-one-out sensitivity analysis to test the potential impact of individual SNPs on the observed causal effect. Furthermore, we evaluated the strength of the IVs selected in our study by calculating F statistic, which allows us to determine the extent to which weak instrument bias may affect our estimates of the causal associations [41]. The equation of the F statistic is F= $\frac{{\textrm{R}}^2}{1-{\textrm{R}}^2}\times \frac{\textrm{n}-\textrm{k}-1}{\textrm{k}}$, where R^2^ represents the proportion of variance explained by SNPs, *n* is the sample size, and *k* is the number of included IVs [42]. R^2^ was estimated by MAF and β value, using the formula: R^2^ = 2 × MAF × (1 − MAF) × β^2^. An F-statistic less than 10 indicates the presence of weak instrumental bias. Finally, we conducted a reverse MR analysis to explore whether asthma has any causal effect on gut microbiota or gut metabolites. The procedure was consistent with the above protocol for the two-sample MR. All statistical analyses were implemented using R software (version 4.1.2) with the R package TwosampleMR (version 0.5.6) and MR-PRESSO (version 1.0).

## Results

### Selection of instrumental variables

After clumping, we selected 128 IVs associated with 9 bacterial taxa for asthma, 130 IVs associated with 12 bacterial taxa for obesity related asthma, 66 IVs associated with 6 bacterial taxa for non-allergic asthma, 55 IVs associated with 4 bacterial taxa for allergic asthma and 59 IVs associated with 5 bacterial taxa for eosinophilic asthma. The F statistics of IVs were all larger than 10, indicating no weak instrumental variables bias. Details about the selected instrumental variables are shown in Additional file 2.

### Causal effects of gut microbiota on asthma

**Fig. 2:**
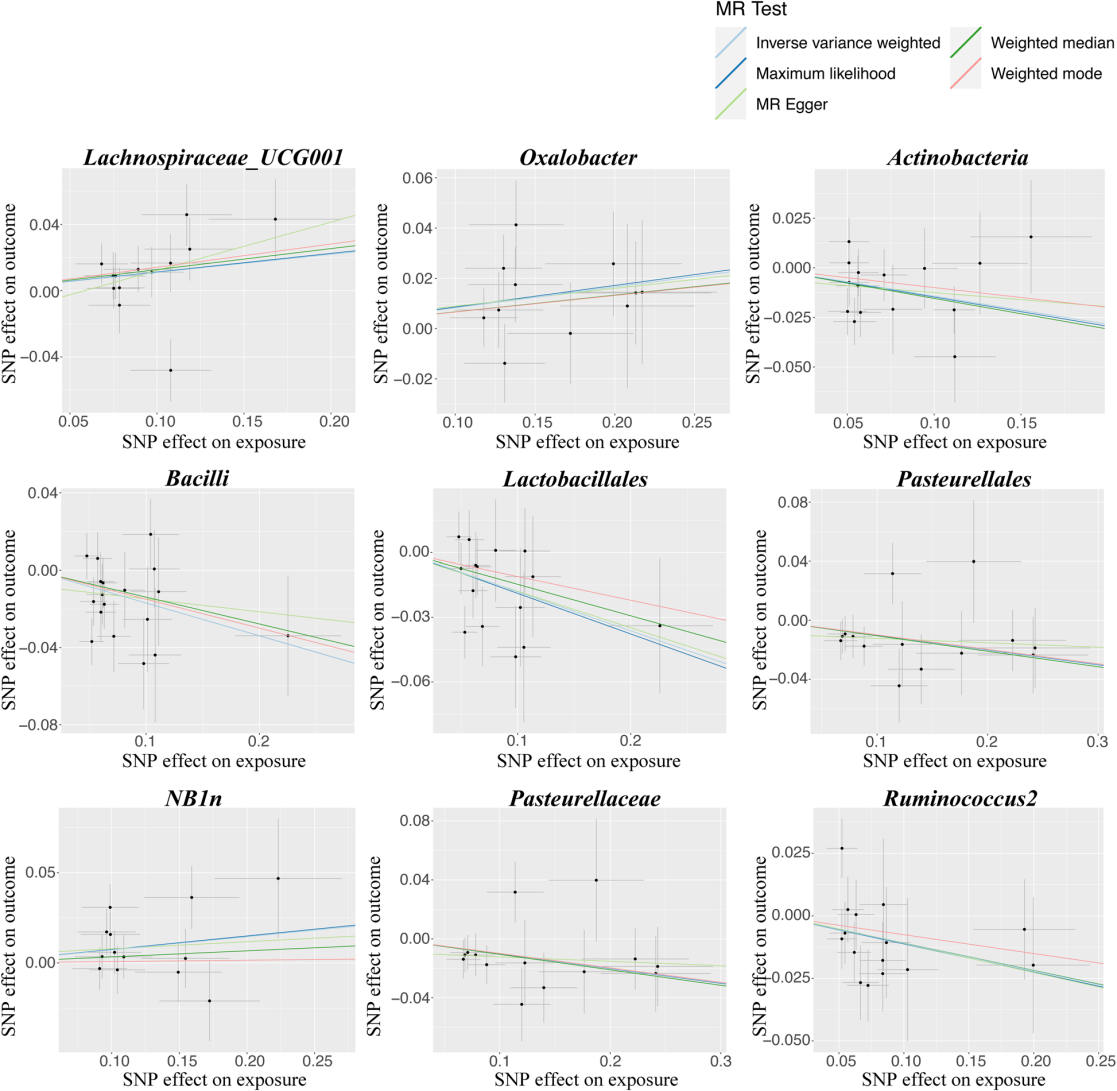
Scatter plots for the causal association between gut microbiota and asthma

As shown in Table 1, the results of IVW analyses indicated that the genetically predicted relative abundance of genus *Lachnospiraceae_UCG001*, genus *Butyricimonas*, and genus *Oxalobacter* were causally associated with a higher risk of asthma, while class *Actinobacteria*, class *Bacilli*, family *Pasteurellaceae*, genus *Ruminococcus2*, order *Lactobacillales*, order *NB1n*, and order *Pasteurellales* were associated with a lower risk of asthma. Except for the causal association between genus *Butyricimonas* and asthma, all other nine causal associations are validated by all five MR analyses, which produced consistent direction of effect estimates (Additional file 1: Table S2). Figure 2 showed scatter plots across various tests. After FDR correction, the IVW estimate of class *Bacilli* (OR = 0.84, 95%CI = 0.76–0.94, *p* = 1.98 × 10^−3^) and order *Lactobacillales* (OR = 0.83, 95%CI = 0.74–0.94, *p* = 1.92 × 10^−3^) remained significantly associated with asthma.

**Table 1:** Causal associations between gut microbiota and asthma by using the IVW method

Exposure	Outcome	Nsnp	Beta	SE	OR	95%CI	***P***
Class *Actinobacteria*	Asthma	15	−0.14	0.05	0.87	0.78–0.96	6.06 × 10^−3^
Class *Bacilli*	Asthma	18	−0.17	0.05	0.84	0.76–0.94	**1.98 × 10**^**−3**^
Family *Pasteurellaceae*	Asthma	14	−0.10	0.04	0.91	0.84–0.98	1.30 × 10^−2^
genus *Butyricimonas*	Asthma	13	0.14	0.06	1.16	1.03–1.29	1.26 × 10^−2^
Genus *Lachnospiraceae_UCG001*	Asthma	13	0.11	0.05	1.12	1.00–1.24	4.63 × 10^−2^
Genus *Oxalobacter*	Asthma	11	0.08	0.03	1.09	1.02–1.16	1.51 × 10^−2^
Genus *Ruminococcus*	Asthma	15	−0.11	0.05	0.90	0.81–0.99	3.02 × 10^−2^
Order *Lactobacillales*	Asthma	15	−0.18	0.06	0.83	0.74–0.94	**1.92 × 10**^**−3**^
Order *NB1n*	Asthma	13	0.07	0.03	1.08	1.01–1.15	3.51 × 10^−2^
Order *Pasteurellales*	Asthma	14	−0.10	0.04	0.91	0.84–0.98	1.30 × 10^−2^

*IVW* inverse-variance weighted: *SNP* single nucleotide polymorphism: *Nsnp* number of SNPs: *SE* standard error: *OR* odds ratio: *CI* confidence interval. *P*-values have undergone FDR correction, with the significance threshold at each taxonomic level calculated as 0.05 divided by the effective number of independent tests (phylum p = 0.05/9, class *p* = 0.05/16, order p = 0.05/20, family p = 0.05/35, and genus p = 0.05/131). *P*-values highlighted in bold denote significant associations, whereas non-bolded values indicate suggestive associations

We further examined the causal associations between gut microbiota and four phenotypes of asthma by the same process. MR analyses identified a total of 27 causal associations, including 12 gut microbiota taxa with obesity related asthma, 6 gut microbiota taxa with non-allergic asthma, 4 bacterial taxa with allergic asthma, and 5 bacterial taxa with eosinophilic asthma (Additional file 1: Table S3). After FDR correction, only the IVW estimate of class *Deltaproteobacteria* (OR = 0.48, 95%CI = 0.31–0.73, *p* = 5.40 × 10^−4^) showed a protective effect against eosinophilic asthma, while other causal associations were no longer significant.

### Causal effects of asthma on gut microbiota

The Reverse MR analysis showed nine causal associations between the risk of asthma on gut microbiota. As shown in Table 2, the results of IVW analyses indicated that the genetically predicted risk of asthma was negatively correlated with the relative abundance of family *Family_XIII*, genus *Anaerostipes*, genus *Eubacterium_xylanophilum_group*, genus *Family_XIII_UCG001*, genus *Lachnospiraceae_NK4A136_group*, genus *Marvinbryantia*, and genus *Ruminococcus_torques_group*, while it was positively correlated with the relative abundance of genus *Anaerofilum*, genus *Intestinimonas*, genus *Lachnospiraceae_UCG004*, and genus *Lachnospira.* Except for the genus *Eubacterium_xylanophilum_group* and genus *Intestinimonas*, all other nine causal associations were validated by all five MR analyses (Additional file 1: Table S4). Scatter plots across various tests are displayed in Additional file 1: Figure S1. However, these associations were no longer significant after FDR correction.

**Table 2:** Causal associations between asthma and gut microbiota by using the IVW method

Exposure	Outcome	Nsnp	Beta	SE	OR	95%CI	***P***
Asthma	Family *Family_XIII*	61	−0.05	0.02	0.95	0.91–0.99	2.60 × 10^−2^
Asthma	Genus *Anaerofilum*	59	0.10	0.04	1.10	1.02–1.19	1.56 × 10^−2^
Asthma	Genus *Anaerostipes*	61	−0.05	0.02	0.95	0.91–1.00	3.33 × 10^−2^
Asthma	genus *Eubacterium_xylanophilum_group*	60	−0.07	0.03	0.93	0.88–0.99	1.32 × 10^−2^
Asthma	Genus *Family_XIII_UCG001*	60	−0.07	0.03	0.93	0.89–0.98	5.54 × 10^−3^
Asthma	genus *Intestinimonas*	60	0.05	0.03	1.05	1.00–1.11	4.74 × 10^−2^
Asthma	Genus *Lachnospiraceae_NK4A136_Group*	61	−0.04	0.02	0.96	0.92–1.00	3.77 × 10^−2^
Asthma	Genus *Lachnospiraceae_UCG004*	60	0.05	0.02	1.05	1.01–1.11	2.92 × 10^−2^
Asthma	Genus *Lachnospira*	5	0.27	0.12	1.32	1.04–1.66	2.20 × 10^−2^
Asthma	Genus *Marvinbryantia*	60	−0.08	0.03	0.93	0.88–0.98	4.13 × 10^−3^
Asthma	Genus *Ruminococcus_Torques_Group*	61	−0.05	0.02	0.95	0.91–0.99	2.77 × 10^−2^

*IVW* inverse-variance weighted, *SNP* single nucleotide polymorphism, *Nsnp* number of SNPs, *SE* standard error, *OR* odds ratio, *CI* confidence interval. *P*-values have undergone FDR correction, with the significance threshold at each taxonomic level calculated as 0.05 divided by the effective number of independent tests (phylum p = 0.05/9, class *p* = 0.05/16, order p = 0.05/20, family p = 0.05/35, and genus p = 0.05/131). *P*-values highlighted in bold denote significant associations, whereas non-bolded values indicate suggestive associations

Causal associations between four phenotypes of asthma and gut microbiota were also analyzed by the same process. A total of 18 causal associations were identified, including obesity related asthma with 3 gut microbiota taxa, non-allergic asthma with 6 gut microbiota taxa, allergic asthma with 2 bacterial taxa, and eosinophilic asthma with 7 bacterial taxa (Additional file 1: Table S5). However, these associations were no longer significant after FDR correction.

### Bi-directional MR analysis of gut metabolites and asthma

Similar to the MR analysis of gut microbiota and asthma, five MR methods were used to estimate the potential causal relationship between gut metabolites and asthma. There was no significant causality between gut metabolites and the risk of asthma or its phenotypes (all *p* > 0.05) (Additional file 3: Table S6). For reverse MR analysis, we found that only the genetically predicted risk of eosinophilic asthma had a causal association with lower levels of indolepropionate (*p* = 3.81× 10^−2^), and sensitivity analysis results supported the robustness of the MR analysis, as shown in Table 3 and Fig. 2. The MR results of asthma and its four phenotypes did not show causal associations with other gut metabolites (all *p* > 0.05) (Additional file 3: Table S7).

**Table 3:** MR results of causal links between eosinophilic asthma and Indolepropionate

Exposure	Outcome	Method	Nsnp	Beta	SE	***p***	Heterogeneity		Horizontal pleiotropy		
							Q	***p***	Intercept	SE	***p***
Eosinophilic asthma	Indolepropionate	IVW	15	−8.94 × 10^−3^	4.31 × 10^−3^	0.04	6.91	0.91	3.40 × 10^−4^	5.90 × 10^−3^	0.96
Eosinophilic asthma	Indolepropionate	MR Egger	15	−1.01 × 10^−2^	2.14 × 10^−2^	0.64	6.92	0.94			
Eosinophilic asthma	Indolepropionate	Maximum likelihood	15	−9.00 × 10^−3^	4.36 × 10^−3^	0.04					
Eosinophilic asthma	Indolepropionate	Weighted median	15	−9.78 × 10^−3^	5.63 × 10^−3^	0.08					
Eosinophilic asthma	Indolepropionate	Weighted mode	15	−7.33 × 10^−3^	8.03 × 10^−3^	0.38					

*MR* Mendelian randomization: *IVW* inverse-variance weighted: *SE* standard error

### Sensitivity analysis

The MR results underwent pleiotropy and heterogeneity test to further validate the causal associations (Additional file 1: Table S8 and S9). The MR Egger intercept and MR-PRESSO analysis did not reveal any clear evidence of pleiotropy (all *p* > 0.05), and no evidence of heterogeneity was identified according to the Cochran’s Q statistics test (all *p* > 0.05). Additionally, the leave-one-out analysis indicated that no single SNP drives the identified causal associations (Additional file 1: Figure S2 and S3).

## Discussion

In the present study, we conducted MR analyses to examine the causal associations between gut microbiota, metabolites and asthma. Our analysis utilized summary data from the MiBioGen consortium’s largest GWAS meta-analysis of gut microbiota and the FinnGen consortium’s asthma summary data. Our results provide evidence for the causal effects of specific microbiota on asthma and its phenotypes, as well as for reverse causality. Additionally, the risk of eosinophilic asthma was also potentially associated with the lower indolepropionate. To our knowledge, this study represents the first comprehensive MR analysis to explore the potential role of gut microbiota and metabolites in the development of asthma at the gene prediction level, which may contribute to strengthening the theoretical basis for the “gut-lung” axis.

Several studies have reported a potential link between asthma and dysbiosis or altered microbiota in the gut [43–46]. The differences in microbial diversity and composition between healthy individuals and asthma patients indicate a potential involvement of gut microbiota in the development of asthma [47, 48]. However, there is no clear causal relationship between gut microbiota dysbiosis and asthma risk. The use of glucocorticoids in asthma patients may cause alterations in the gut microbiome, and differences in gender ratios and ethnicities between studies may affect the composition of the gut microbiome [49–51]. Furthermore, while studies have found that asthma patients tend to have a phenotype of gut microbiome dysbiosis [52], the results regarding changes in specific strains have been inconsistent, making it difficult to infer a causal link between gut microbiota and asthma risk.

In our study, we aimed to identify specific gut microbiota that are causally associated with asthma and its phenotypes. We identified 47 potential candidates, of which three showed a significant causal relationship with asthma. Our study found that the class *Bacilli* and order *Lactobacillales* were associated with a lower risk of asthma. This finding aligns with existing research, such as the study by Spacova et al., which demonstrated the beneficial effects of *Lactobacillus rhamnosus*, a member of *Lactobacillales*, in preventing airway function deterioration in a murine asthma model [53]. Our results contribute to the growing body of evidence on the role of specific microbiota, including *Lactobacillales*, in asthma pathogenesis.

Our study revealed that the relative abundance of genus *Lachnospiraceae_UCG001* was suggestive causally associated with a higher risk of asthma, while the genetically predicted risk of asthma was positively correlated with the relative abundance of genus *Lachnospiraceae_UCG004*. These findings are consistent with a previous study that has shown increased levels of *Lachnospiraceae* in allergic subjects [54]. However, the direction of associations between *Lachnospiraceae* and asthma has not been consistent. We also observed a negative correlation between the genetically predicted risk of asthma and the relative abundance of the genus *Lachnospiraceae_NK4A136_group*. Similarly, *Lachnospiraceae* has been found to be associated with a decreased risk of eczema and inhalant allergic sensitization [55]. A recent study has also manifested that decreased levels of *Lachnospiraceae* in infancy are associated with allergic disease [56]. Our study suggests that inconsistencies in previous clinical studies may be due to insufficient classification of the genera level of gut microbiota. Notably, members of the *Lachnospiraceae* family have been found to encode B cell “superantigens” that stimulate potent IgA responses resulting in bacterial IgA coating [57]. As major producers of short-chain fatty acids, *Lachnospiraceae* are involved in regulatory T cell development in the gut, and gut regulatory T cells [58], perhaps through IL-10 expression, may be protective against the development of asthma. The *Lachnospiraceae* family includes three main genera: *Ruminococcus, Lachnospira*, and *Anaerofilum*. Arrieta et al. conducted a study on the gut microbiome of infants at risk for asthma in the Canadian Healthy Infant Longitudinal Development (CHILD) Study [59]. They reported a significant decrease in the relative abundance of the genus *Lachnospira* in children at risk of asthma, which was also confirmed in a mouse model of experimental asthma [60]. The authors suggested that inoculation of germ-free mice with these bacterial taxa ameliorated lung inflammation in their adult progeny. Subsequently, another study extended their previous work and found a reduction in the abundance of *Lachnospira* in the 3-month fecal microbiota of asthmatic children, which was considered a potential indicator of asthma diagnosed in preschool-age children [61]. This reduction was accompanied by reduced levels of fecal acetate and dysregulation of enterohepatic metabolites [62]. However, our findings were inconsistent with those studies as we found that asthma was positively correlated with the relative abundance of genus *Lachnospira.* We hypothesize that the positive and negative effects of *Lachnospira* on asthma may be species- and strain-specific, and our study only analyzed data from adults. In addition to *Lachnospiraceae*, we also found that genus *Ruminococcus2* was significantly causally associated with a lower risk of asthma, while the genetically predicted risk of asthma was negatively correlated with the relative abundance of genus *Ruminococcus_torques_group*, suggesting that genus *Ruminococcus* may have a protective effect against asthma. These findings are in line with previous research that has shown a low relative abundance of the genus *Ruminococcus* in stools collected during early childhood is linked to an increased risk of asthma [63]. In addition, a reduction of *Ruminococcus* was also negatively correlated with the total fecal IgE levels and strongly associated with children who have mite-sensitized asthma [64]. Furthermore, our study revealed suggestive causal effects of genus *Oxalobacter on* a higher risk of asthma, and class *Actinobacteria*, family *Pasteurellaceae*, order *NB1n*, and order *Pasteurellales* on a lower risk of asthma. Prior studies, including Chung KF [65] and Perez-Garcia et al. [66], have highlighted the involvement of *Actinobacteria* and *Pasteurellaceae* in asthma risk, indicating these taxa’s potential as biomarkers and therapeutic targets. Our study contributes to the understanding of these relationships by quantifying their effects on asthma risk and underscores the complexity of the microbiome’s role in respiratory health.

We conducted an analysis to examine the possible associations between gut metabolites and asthma, as they play a crucial role in the interplay between gut microbiota and asthma. While previous studies have suggested potential roles for gut metabolites in asthma, our MR study failed to demonstrate the causality of genetically predicted gut microbiota with asthma. However, our study did reveal that eosinophilic asthma was associated with lower levels of indolepropionate (*p* = 3.81 × 10^-2), albeit this *p*-value is nominal and has not been adjusted for multiple comparisons. This finding, therefore, should be viewed as exploratory, prompting further investigation into the role of indolepropionate as a potential biomarker for eosinophilic asthma. Indolepropionate has been shown to activate mouse pregnane X receptor (PXR) and induces anti-inflammatory effects [67]. Previous studies have revealed that a higher level of indolepropionate was associated with a lower risk of type 2 diabetes and increased insulin secretion [68, 69]. Another large population-based study showed that increased physical activity was significantly associated with high levels of indolepropionate [70]. Considering these multifaceted implications, the association of indolepropionate with asthma, particularly eosinophilic asthma, warrants a deeper investigation.

Our MR study presents several noteworthy advantages. Firstly, we employed a distinctive two-sample bi-directional MR design to investigate the potential causal association between gut microbiota and metabolites with asthma, thereby providing a robust theoretical foundation for the “gut-lung axis” mechanisms. Secondly, we utilized one of the largest available GWAS summary datasets, ensuring sufficient statistical power to detect causal effects accurately. Lastly, we comprehensively analyzed four distinct asthma phenotypes, enabling us to evaluate the common gut microbiota causally related to different asthma phenotypes and identify novel insights into the gut microbiota-mediating pathogenesis of asthma.

However, there are also several limitations in this study. Firstly, the study included a relaxed cutoff for instrumental variables selection (*p* < 1 × 10^−5^) for gut microbiota and metabolites due to limited SNPs meeting the genome-wide significance threshold (*p* < 5 × 10^−8^), potentially leading to weak instrument bias. Nonetheless, we addressed these limitations using F-statistics and sensitivity analyses to ensure the validity of the results. Secondly, we only described gut microbiota at the genus level or above due to the lack of data at the species level, highlighting the need for metagenomic sequencing techniques to obtain more specific and accurate results. Thirdly, our study was constrained by the availability of demographic information in the underlying data sources, which precluded us from conducting further subgroup analyses to explore age-specific or gender-specific causal relationships between gut microbiota and asthma. Fourthly, The Finnish population has unique genetic characteristics due to historical events, which may limit the generalizability of our findings to other populations. While this specificity offers valuable insights into asthma genetics, it also means that our results may not fully represent the genetic associations found in more genetically diverse populations. Fifthly, MiBioGen, predominantly of European ancestry, includes approximately 28% with other/multiple ancestries, while all FinnGen individuals are of European descent. We acknowledge that varying genetic ancestries can lead to differences in LD patterns, potentially influencing the robustness of our MR results. Furthermore, the gut metabolites GWAS in our study had a relatively small sample size and limited loci studied. Therefore, further research with larger GWAS statistics is necessary to provide a more precise evaluation of the association between gut metabolites and asthma. Additionally, we recognize a limitation regarding the MR assumption of genotype independence from microbe-asthma confounders. Anthropomorphic traits like BMI, which influence both microbial abundance and asthma risk and have genetic components, might introduce unmeasured confounding in our analysis.

## Conclusions

In summary, our MR study provides compelling evidence supporting a causal relationship between the gut microbiota on the development of asthma. These findings offer novel insights into the underlying mechanisms of microbiota-mediated asthma and highlight the potential for targeted manipulation of the gut microbiota in the prevention and treatment of this disease. However, further studies are necessary to fully understand the mechanisms underlying this relationship and to evaluate the efficacy of gut microbiota manipulation as a therapeutic strategy for asthma.

### Supplementary Information

**Additional file 1: Table S1.** Characteristics of included GWAS summary-level data of gut microbiota, gut metabolites and asthma. **Table S2.** Causal associations between gut microbiota and asthma by using other four methods. **Table S3.** Full result of MR estimates for causal associations between gut microbiota and four phenotypes of asthma. **Table S4.** Causal associations between asthma and gut microbiota by using other four methods. **Table S5.** Full result of MR estimates for causal associations between four phenotypes of asthma and gut microbiota. **Table S8.** The sensitivity analyses of causality between gut microbiota and asthma and its phenotypes based on MR results. **Table S9.** The sensitivity analyses of causality between asthma and its phenotypes and gut microbiota based on MR results. **Figure S1.** Scatter plots for the causal association between asthma and gut microbiota. **Figure S2.** Leave-one-out plots for the causal association between gut microbiota and asthma. **Figure S3.** Leave-one-out plots for the causal association between asthma and gut microbiota.

**Additional file 2:** Detailed information on genetic variants included as instruments for traits.

**Additional file 3: Table S6.** Full result of MR estimates for causal associations between gut metabolites and asthma. **Table S7.** Full result of MR estimates for causal associations between asthma and gut metabolites.

## Data Availability

The GWAS data of gut microbiota were retrieved from GWAS Catalog (https://www.ebi.ac.uk/gwas/publications/33462485). The GWAS data of gut metabolites were retrieved from GWAS Catalog (https://www.ebi.ac.uk/gwas/publications/24816252). The GWAS data of asthma and its phenotypes were retrieved from IEU-OpenGWAS project (https://gwas.mrcieu.ac.uk/datasets/finn-b-J10_ASTHMA/, https://gwas.mrcieu.ac.uk/datasets/finn-b-ASTHMA_OBESITY/, https://gwas.mrcieu.ac.uk/datasets/finn-b-ASTHMA_NONALLERG/, https://gwas.mrcieu.ac.uk/datasets/finn-b-ALLERG_ASTHMA/, and https://gwas.mrcieu.ac.uk/datasets/finn-b-ASTHMA_EOSINOPHIL_SUGG/).

## Abbreviations

MR: Mendelian randomization
IVs: Instrumental variables
GWAS: Genome-wide association studies
SNPs: Single-nucleotide polymorphisms
IVW: Inverse-variance weighted
MR-PRESSO: Mendelian Randomization Pleiotropy RESidual Sum and Outlier
LD: Linkage disequilibrium
MAF: Minor allele frequency
Nsnp: Number of single-nucleotide polymorphisms
SE: Standard error
OR: Odds ratio
CI: Confidence interval
FDR: False discovery rate
CHILD: Canadian Healthy Infant Longitudinal Development
PXR: Pregnane X receptor
