# Supplementary material for: Causal associations between gut microbiota, metabolites and asthma: a two-sample Mendelian randomization study
Source: BMC Pulm Med. 2024 Feb 7;24:72. doi: 10.1186/s12890-024-02898-x (PMC10848467; doi:10.1186/s12890-024-02898-x)
Supplement: Supplementary file 1 — Additional file 1: Table S1. Characteristics of included GWAS summary-level data of gut microbiota, gut metabolites and asthma. Table S2. Causal associations between gut microbiota and asthma by using other four methods. Table S3. Full result of MR estimates for causal associations between gut microbiota and four phenotypes of asthma. Table S4. Causal associations between asthma and gut microbiota by using other four methods. Table S5. Full result of MR estimates for causal associations between four phenotypes of asthma and gut microbiota. Table S8. The sensitivity analyses of causality between gut microbiota and asthma and its phenotypes based on MR results. Table S9. The sensitivity analyses of causality between asthma and its phenotypes and gut microbiota based on MR results. Figure S1. Scatter plots for the causal association between asthma and gut microbiota. Figure S2. Leave-one-out plots for the causal association between gut microbiota and asthma. Figure S3. Leave-one-out plots for the causal association between asthma and gut microbiota. [file 12890_2024_2898_MOESM1_ESM.docx]

## Contents

**Additional file 1:  Table S1.**Characteristics of included GWAS summary-level data of gut microbiota, gut metabolites and asthma. **Table S2.**Causal associations between gut microbiota and asthma by using other four methods. **Table S3.** Full result of MR estimates for causal associations between gut microbiota and four phenotypes of asthma. **Table S4.** Causal associations between asthma and gut microbiota by using other four methods. **Table S5.** Full result of MR estimates for causal associations between four phenotypes of asthma and gut microbiota. **Table S8.**The sensitivity analyses of causality between gut microbiota and asthma and its phenotypes based on MR results. **Table S9.** The sensitivity analyses of causality between asthma and its phenotypes and gut microbiota based on MR results. **Figure S1.** Scatter plots for the causal association between asthma and gut microbiota. **Figure S2.** Leave-one-out plots for the causal association between gut microbiota and asthma. **Figure S3.** Leave-one-out plots for the causal association between asthma and gut microbiota.

**Additional file 2:** Detailed information on genetic variants included as instruments for traits.

**Additional file 3: Table S6.** Full result of MR estimates for causal associations between gut metabolites and asthma. **Table S7.** Full result of MR estimates for causal associations between asthma and gut metabolites.

Table S1. Characteristics of included GWAS summary-level data of gut microbiota, gut metabolites and asthma

| **Trait** | **Consortium** | **Sample size** | **Case** | **Control** | **Population** | **Year** |
| --- | --- | --- | --- | --- | --- | --- |
| Gut microbiota | MiBioGen | 18340 | NA | NA | Mixed | 2021 |
| Gut metabolites | TwinsUK and KORA | 7284 | NA | NA | European | 2013 |
| Asthma | FinnGen | NA | 20629 | 135449 | European | 2021 |
| Obesity related asthma | FinnGen | NA | 4142 | 135449 | European | 2021 |
| Non-allergic asthma | FinnGen | NA | 3155 | 135449 | European | 2021 |
| Allergic asthma | FinnGen | NA | 4859 | 135449 | European | 2021 |
| Eosinophilic asthma | FinnGen | NA | 1184 | 135449 | European | 2021 |

Table S2. Causal associations between gut microbiota and asthma by using other four methods.

| **Exposure** | **Outcome** | **Method** | **Beta** | **SE** | **OR** | **95%CI** | ***P*** |
| --- | --- | --- | --- | --- | --- | --- | --- |
| Class *Actinobacteria* | Asthma | Maximum likelihood | -0.15 | 0.05 | 0.86 | 0.78-0.96 | 5.24 × 10^-3^ |
|  | Asthma | MR Egger | -0.07 | 0.15 | 0.93 | 0.69-1.26 | 6.54 × 10^-1^ |
|  | Asthma | Weighted median | -0.15 | 0.07 | 0.86 | 0.74-0.99 | 3.77 × 10^-2^ |
|  | Asthma | Weighted mode | -0.10 | 0.10 | 0.91 | 0.75-1.10 | 3.26 × 10^-1^ |
| Class *Bacilli* | Asthma | Maximum likelihood | -0.17 | 0.06 | 0.84 | 0.76-0.94 | 2.03 × 10^-3^ |
|  | Asthma | MR Egger | -0.07 | 0.15 | 0.94 | 0.69-1.26 | 6.69 × 10^-1^ |
|  | Asthma | Weighted median | -0.14 | 0.08 | 0.87 | 0.74-1.02 | 8.81 × 10^-2^ |
|  | Asthma | Weighted mode | -0.15 | 0.13 | 0.86 | 0.67-1.11 | 2.57 × 10^-1^ |
| Family *Pasteurellaceae* | Asthma | Maximum likelihood | -0.10 | 0.04 | 0.90 | 0.83-0.98 | 1.48 × 10^-2^ |
|  | Asthma | MR Egger | -0.03 | 0.09 | 0.97 | 0.82-1.15 | 7.36 × 10^-1^ |
|  | Asthma | Weighted median | -0.11 | 0.05 | 0.90 | 0.81-1.00 | 5.61 × 10^-2^ |
|  | Asthma | Weighted mode | -0.10 | 0.07 | 0.91 | 0.80-1.03 | 1.63 × 10^-1^ |
| Genus *Lachnospiraceae_UCG001* | Asthma | Maximum likelihood | 0.11 | 0.05 | 1.12 | 1.02-1.23 | 1.85 × 10^-2^ |
|  | Asthma | MR Egger | 0.29 | 0.24 | 1.34 | 0.83-2.15 | 2.51 × 10^-1^ |
|  | Asthma | Weighted median | 0.13 | 0.06 | 1.14 | 1.00-1.28 | 4.44 × 10^-2^ |
|  | Asthma | Weighted mode | 0.14 | 0.09 | 1.15 | 0.97-1.37 | 1.43 × 10^-1^ |
| Genus *Oxalobacter* | Asthma | Maximum likelihood | 0.09 | 0.04 | 1.09 | 1.02-1.17 | 1.41 × 10^-2^ |
|  | Asthma | MR Egger | 0.07 | 0.16 | 1.07 | 0.78-1.47 | 6.70 × 10^-1^ |
|  | Asthma | Weighted median | 0.07 | 0.05 | 1.07 | 0.98-1.17 | 1.44 × 10^-1^ |
|  | Asthma | Weighted mode | 0.07 | 0.07 | 1.07 | 0.93-1.23 | 3.75 × 10^-1^ |
| Genus *Ruminococcus2* | Asthma | Maximum likelihood | -0.11 | 0.05 | 0.89 | 0.81-0.99 | 2.74 × 10^-2^ |
|  | Asthma | MR Egger | -0.12 | 0.13 | 0.89 | 0.69-1.14 | 3.78 × 10^-1^ |
|  | Asthma | Weighted median | -0.11 | 0.07 | 0.90 | 0.78-1.03 | 1.12 × 10^-1^ |
|  | Asthma | Weighted mode | -0.08 | 0.08 | 0.93 | 0.79-1.09 | 3.84 × 10^-1^ |
| Order *Lactobacillales* | Asthma | Maximum likelihood | -0.11 | 0.05 | 0.89 | 0.81-0.99 | 2.74 × 10^-2^ |
|  | Asthma | MR Egger | -0.17 | 0.16 | 0.84 | 0.62-1.15 | 3.01 × 10^-1^ |
|  | Asthma | Weighted median | -0.15 | 0.08 | 0.86 | 0.73-1.02 | 7.92 × 10^-2^ |
|  | Asthma | Weighted mode | -0.11 | 0.11 | 0.90 | 0.72-1.11 | 3.29 × 10^-1^ |
| Order *NB1n* | Asthma | Maximum likelihood | 0.07 | 0.03 | 1.08 | 1.01-1.15 | 3.23 × 10^-2^ |
|  | Asthma | MR Egger | 0.04 | 0.15 | 1.04 | 0.77-1.40 | 8.04 × 10^-1^ |
|  | Asthma | Weighted median | 0.03 | 0.05 | 1.03 | 0.94-1.14 | 4.81 × 10^-1^ |
|  | Asthma | Weighted mode | 0.01 | 0.07 | 1.01 | 0.87-1.17 | 9.20 × 10^-1^ |
| Order *Pasteurellales* | Asthma | Maximum likelihood | -0.10 | 0.04 | 0.90 | 0.83-0.98 | 1.48 × 10^-2^ |
|  | Asthma | MR Egger | -0.03 | 0.09 | 0.97 | 0.82-1.15 | 7.36 × 10^-1^ |
|  | Asthma | Weighted median | -0.11 | 0.05 | 0.90 | 0.81-1.00 | 4.70 × 10^-2^ |
|  | Asthma | Weighted mode | -0.10 | 0.07 | 0.91 | 0.80-1.03 | 1.57 × 10^-1^ |

MR, Mendelian randomization; OR, odds ratio; CI, confidence interval.

Table S3. Full result of MR estimates for causal associations between gut microbiota and four phenotypes of asthma

| **Exposure** | **Outcome** | **Method** | **Nsnp** | **Beta** | **SE** | **OR** | **95%CI** | ***P*** |
| --- | --- | --- | --- | --- | --- | --- | --- | --- |
| Class *Deltaproteobacteria* | Obesity related asthma | IVW | 13 | -0.29 | 0.11 | 0.75 | 0.60-0.93 | 9.14 × 10^-3^ |
|  |  | Maximum likelihood | 13 | -0.30 | 0.12 | 0.74 | 0.59-0.93 | 9.99 × 10^-3^ |
|  |  | MR Egger | 13 | -0.27 | 0.30 | 0.77 | 0.43-1.38 | 3.94 × 10^-1^ |
|  |  | Weighted median | 13 | -0.29 | 0.16 | 0.75 | 0.55-1.01 | 6.12 × 10^-2^ |
|  |  | Weighted mode | 13 | -0.27 | 0.22 | 0.76 | 0.50-1.17 | 2.36 × 10^-1^ |
| Family *Family_XIII* | Obesity related asthma | IVW | 9 | -0.32 | 0.15 | 0.73 | 0.54-0.98 | 3.78 × 10^-2^ |
|  |  | Maximum likelihood | 9 | -0.33 | 0.16 | 0.72 | 0.53-0.98 | 3.91 × 10^-2^ |
|  |  | MR Egger | 9 | -0.15 | 0.62 | 0.86 | 0.26-2.89 | 8.17 × 10^-1^ |
|  |  | Weighted median | 9 | -0.40 | 0.21 | 0.67 | 0.44-1.02 | 5.92 × 10^-2^ |
|  |  | Weighted mode | 9 | -0.54 | 0.34 | 0.58 | 0.30-1.12 | 1.46 × 10^-1^ |
| Family *Pasteurellaceae* | Obesity related asthma | IVW | 14 | -0.24 | 0.09 | 0.79 | 0.66-0.94 | 7.40 × 10^-3^ |
|  |  | Maximum likelihood | 14 | -0.25 | 0.08 | 0.78 | 0.66-0.92 | 3.31 × 10^-3^ |
|  |  | MR Egger | 14 | -0.13 | 0.20 | 0.88 | 0.59-1.30 | 5.23 × 10^-1^ |
|  |  | Weighted median | 14 | -0.19 | 0.11 | 0.82 | 0.66-1.03 | 8.28 × 10^-2^ |
|  |  | Weighted mode | 14 | -0.27 | 0.13 | 0.76 | 0.59-0.99 | 5.96 × 10^-2^ |
| Family *Rikenellaceae* | Obesity related asthma | IVW | 17 | -0.27 | 0.11 | 0.76 | 0.62-0.95 | 1.32 × 10^-2^ |
|  |  | Maximum likelihood | 17 | -0.26 | 0.11 | 0.77 | 0.62-0.95 | 1.74 × 10^-2^ |
|  |  | MR Egger | 17 | -0.53 | 0.34 | 0.59 | 0.30-1.14 | 1.37 × 10^-1^ |
|  |  | Weighted median | 17 | -0.21 | 0.15 | 0.81 | 0.60-1.09 | 1.58 × 10^-1^ |
|  |  | Weighted mode | 17 | -0.05 | 0.26 | 0.95 | 0.57-1.57 | 8.38 × 10^-1^ |
| Genus *Anaerofilum* | Obesity related asthma | IVW | 11 | -0.17 | 0.08 | 0.84 | 0.72-0.98 | 2.50 × 10^-2^ |
|  |  | Maximum likelihood | 11 | -0.18 | 0.08 | 0.84 | 0.72-0.98 | 2.54 × 10^-2^ |
|  |  | MR Egger | 11 | -0.67 | 0.42 | 0.51 | 0.23-1.17 | 1.46 × 10^-1^ |
|  |  | Weighted median | 11 | -0.09 | 0.10 | 0.91 | 0.75-1.10 | 3.41 × 10^-1^ |
|  |  | Weighted mode | 11 | -0.06 | 0.14 | 0.94 | 0.71-1.23 | 6.54 × 10^-1^ |
| Genus *Collinsella* | Obesity related asthma | IVW | 9 | -0.36 | 0.15 | 0.70 | 0.52-0.94 | 1.68 × 10^-2^ |
|  |  | Maximum likelihood | 9 | -0.37 | 0.15 | 0.69 | 0.51-0.94 | 1.70 × 10^-2^ |
|  |  | MR Egger | 9 | -0.34 | 0.56 | 0.71 | 0.24-2.14 | 5.66 × 10^-1^ |
|  |  | Weighted median | 9 | -0.25 | 0.20 | 0.78 | 0.52-1.15 | 2.09 × 10^-1^ |
|  |  | Weighted mode | 9 | -0.22 | 0.28 | 0.81 | 0.47-1.38 | 4.57 × 10^-1^ |
| Genus *Ruminococcaceae_UCG010* | Obesity related asthma | IVW | 6 | -0.34 | 0.16 | 0.71 | 0.52-0.98 | 3.51 × 10^-2^ |
|  |  | Maximum likelihood | 6 | -0.36 | 0.16 | 0.70 | 0.52-0.95 | 2.22 × 10^-2^ |
|  |  | MR Egger | 6 | -0.50 | 0.49 | 0.60 | 0.23-1.59 | 3.66 × 10^-1^ |
|  |  | Weighted median | 6 | -0.33 | 0.20 | 0.72 | 0.48-1.07 | 1.00 × 10^-1^ |
|  |  | Weighted mode | 6 | -0.32 | 0.24 | 0.72 | 0.45-1.17 | 2.43 × 10^-1^ |
| Genus *Terrisporobacter* | Obesity related asthma | IVW | 5 | 0.27 | 0.13 | 1.31 | 1.01-1.70 | 4.34 × 10^-2^ |
|  |  | Maximum likelihood | 5 | 0.28 | 0.14 | 1.32 | 1.00-1.73 | 4.63 × 10^-2^ |
|  |  | MR Egger | 5 | 0.66 | 0.40 | 1.94 | 0.88-4.26 | 1.99 × 10^-1^ |
|  |  | Weighted median | 5 | 0.21 | 0.17 | 1.23 | 0.88-1.74 | 2.25 × 10^-1^ |
|  |  | Weighted mode | 5 | 0.21 | 0.21 | 1.23 | 0.82-1.85 | 3.75 × 10^-1^ |
| Genus *Turicibacter* | Obesity related asthma | IVW | 10 | -0.22 | 0.10 | 0.81 | 0.66-0.98 | 3.39 × 10^-2^ |
|  |  | Maximum likelihood | 10 | -0.21 | 0.10 | 0.81 | 0.66-0.99 | 3.85 × 10^-2^ |
|  |  | MR Egger | 10 | -0.95 | 0.44 | 0.39 | 0.16-0.90 | 5.98 × 10^-2^ |
|  |  | Weighted median | 10 | -0.27 | 0.14 | 0.76 | 0.59-1.00 | 4.93 × 10^-2^ |
|  |  | Weighted mode | 10 | -0.29 | 0.18 | 0.75 | 0.53-1.06 | 1.35 × 10^-1^ |
| Order *Bacillales* | Obesity related asthma | IVW | 9 | 0.15 | 0.07 | 1.16 | 1.00-0.98 | 4.95 × 10^-2^ |
|  |  | Maximum likelihood | 9 | 0.15 | 0.07 | 1.17 | 1.02-1.33 | 2.43 × 10^-2^ |
|  |  | MR Egger | 9 | 0.05 | 0.34 | 1.05 | 0.5402.03 | 8.93 × 10^-1^ |
|  |  | Weighted median | 9 | 0.23 | 0.09 | 1.25 | 1.05-1.50 | 1.33 × 10^-2^ |
|  |  | Weighted mode | 9 | 0.29 | 0.20 | 1.34 | 0.91-1.97 | 1.75 × 10^-1^ |
| Order *NB1n* | Obesity related asthma | IVW | 13 | 0.18 | 0.07 | 1.20 | 1.05-1.37 | 8.21 × 10^-3^ |
|  |  | Maximum likelihood | 13 | 0.18 | 0.07 | 1.20 | 1.05-1.38 | 7.99 × 10^-3^ |
|  |  | MR Egger | 13 | 0.12 | 0.29 | 1.13 | 0.64-1.99 | 6.80 × 10^-1^ |
|  |  | Weighted median | 13 | 0.15 | 0.09 | 1.17 | 0.98-1.39 | 8.31 × 10^-2^ |
|  |  | Weighted mode | 13 | 0.13 | 0.14 | 1.14 | 0.86-1.49 | 3.83 × 10^-1^ |
| Order *Pasteurellales* | Obesity related asthma | IVW | 14 | -0.24 | 0.09 | 0.79 | 0.66-0.94 | 7.40 × 10^-3^ |
|  |  | Maximum likelihood | 14 | -0.25 | 0.08 | 0.78 | 0.66-0.92 | 3.31 × 10^-3^ |
|  |  | MR Egger | 14 | -0.13 | 0.20 | 0.88 | 0.59-1.30 | 5.23 × 10^-1^ |
|  |  | Weighted median | 14 | -0.19 | 0.11 | 0.82 | 0.66-1.02 | 7.90 × 10^-2^ |
|  |  | Weighted mode | 14 | -0.27 | 0.14 | 0.76 | 0.59-0.99 | 6.68 × 10^-2^ |
| Class *Bacilli* | Non-allergic asthma | IVW | 18 | -0.24 | 0.11 | 0.79 | 0.63-0.98 | 3.33 × 10^-2^ |
|  |  | Maximum likelihood | 18 | -0.23 | 0.11 | 0.79 | 0.64-0.98 | 3.47 × 10^-2^ |
|  |  | MR Egger | 18 | -0.59 | 0.31 | 0.55 | 0.30-1.01 | 7.23 × 10^-2^ |
|  |  | Weighted median | 18 | -0.29 | 0.16 | 0.75 | 0.55-1.01 | 6.06 × 10^-2^ |
|  |  | Weighted mode | 18 | -0.43 | 0.29 | 0.65 | 0.37-1.14 | 1.54 × 10^-1^ |
| Family *Acidaminococcaceae* | Non-allergic asthma | IVW | 7 | -0.36 | 0.16 | 0.70 | 0.51-0.98 | 2.30 × 10^-2^ |
|  |  | Maximum likelihood | 7 | -0.38 | 0.14 | 0.69 | 0.52-0.90 | 6.70 × 10^-3^ |
|  |  | MR Egger | 7 | -1.02 | 0.42 | 0.36 | 0.16-0.83 | 6.06 × 10^-2^ |
|  |  | Weighted median | 7 | -0.37 | 0.20 | 0.69 | 0.47-1.01 | 5.85 × 10^-2^ |
|  |  | Weighted mode | 7 | -0.52 | 0.28 | 0.59 | 0.35-1.02 | 1.10 × 10^-1^ |
| Genus *Desulfovibrio* | Non-allergic asthma | IVW | 10 | -0.26 | 0.11 | 0.77 | 0.62-0.95 | 1.68 × 10^-2^ |
|  |  | Maximum likelihood | 10 | -0.27 | 0.11 | 0.76 | 0.61-0.95 | 1.66 × 10^-2^ |
|  |  | MR Egger | 10 | -0.52 | 0.34 | 0.60 | 0.30-1.17 | 1.69 × 10^-1^ |
|  |  | Weighted median | 10 | -0.24 | 0.15 | 0.78 | 0.59-1.05 | 1.00 × 10^-1^ |
|  |  | Weighted mode | 10 | -0.24 | 0.22 | 0.79 | 0.51-1.21 | 3.02 × 10^-1^ |
| Genus *Eubacterium_Nodatum_Group* | Non-allergic asthma | IVW | 11 | -0.13 | 0.06 | 0.88 | 0.77-1.00 | 4.24 × 10^-2^ |
|  |  | Maximum likelihood | 11 | -0.13 | 0.07 | 0.87 | 0.77-1.00 | 4.34 × 10^-2^ |
|  |  | MR Egger | 11 | -0.11 | 0.29 | 0.89 | 0.51-1.57 | 7.05 × 10^-1^ |
|  |  | Weighted median | 11 | -0.10 | 0.09 | 0.90 | 0.76-1.08 | 2.55 × 10^-1^ |
|  |  | Weighted mode | 11 | -0.04 | 0.13 | 0.96 | 0.74-1.24 | 7.57 × 10^-1^ |
| Genus *Eubacterium_Xylanophilum_Group* | Non-allergic asthma | IVW | 9 | 0.25 | 0.13 | 1.29 | 1.01-1.65 | 4.47 × 10^-2^ |
|  |  | Maximum likelihood | 9 | 0.26 | 0.13 | 1.30 | 1.01-1.68 | 4.43 × 10^-2^ |
|  |  | MR Egger | 9 | 0.10 | 0.38 | 1.10 | 0.53-2.31 | 8.02 × 10^-1^ |
|  |  | Weighted median | 9 | 0.23 | 0.17 | 1.26 | 0.91-1.75 | 1.66 × 10^-1^ |
|  |  | Weighted mode | 9 | 0.02 | 0.25 | 1.03 | 0.63-1.68 | 9.24 × 10^-1^ |
| Genus *Oxalobacter* | Non-allergic asthma | IVW | 11 | 0.16 | 0.07 | 1.18 | 1.03-1.35 | 2.05 × 10^-2^ |
|  |  | Maximum likelihood | 11 | 0.17 | 0.07 | 1.18 | 1.03-1.37 | 1.96 × 10^-2^ |
|  |  | MR Egger | 11 | 0.19 | 0.34 | 1.21 | 0.62-2.36 | 5.89 × 10^-1^ |
|  |  | Weighted median | 11 | 0.07 | 0.10 | 1.08 | 0.89-1.31 | 4.48 × 10^-1^ |
|  |  | Weighted mode | 11 | 0.00 | 0.17 | 1.00 | 0.72-1.40 | 9.86 × 10^-1^ |
| Class *Bacteroidia* | Allergic asthma | IVW | 14 | -0.28 | 0.11 | 0.75 | 0.61-0.93 | 8.20 × 10^-3^ |
|  |  | Maximum likelihood | 14 | -0.29 | 0.11 | 0.75 | 0.61-0.93 | 7.75 × 10^-3^ |
|  |  | MR Egger | 14 | -0.54 | 0.22 | 0.58 | 0.38-0.89 | 2.84 × 10^-2^ |
|  |  | Weighted median | 14 | -0.25 | 0.15 | 0.78 | 0.58-1.04 | 9.38 × 10^-2^ |
|  |  | Weighted mode | 14 | -0.19 | 0.27 | 0.83 | 0.49-1.49 | 4.95 × 10^-1^ |
| Genus *Ruminococcaceae_UCG009* | Allergic asthma | IVW | 12 | 0.19 | 0.08 | 1.20 | 1.03-1.41 | 2.08 × 10^-2^ |
|  |  | Maximum likelihood | 12 | 0.19 | 0.08 | 1.22 | 1.04-1.43 | 1.71 × 10^-2^ |
|  |  | MR Egger | 12 | 0.54 | 0.31 | 1.72 | 0.93-3.19 | 1.15 × 10^-1^ |
|  |  | Weighted median | 12 | 0.08 | 0.11 | 1.08 | 0.871.35 | 4.72 × 10^-1^ |
|  |  | Weighted mode | 12 | 0.06 | 0.18 | 1.06 | 0.74-1.50 | 7.62 × 10^-1^ |
| Order *Bacteroidales* | Allergic asthma | IVW | 14 | -0.28 | 0.11 | 0.75 | 0.61-0.93 | 8.20 × 10^-3^ |
|  |  | Maximum likelihood | 14 | -0.29 | 0.11 | 0.75 | 0.61-0.93 | 7.75 × 10^-3^ |
|  |  | MR Egger | 14 | -0.54 | 0.22 | 0.58 | 0.38-0.89 | 2.84 × 10^-2^ |
|  |  | Weighted median | 14 | -0.25 | 0.15 | 0.78 | 0.58-1.05 | 1.01 × 10^-1^ |
|  |  | Weighted mode | 14 | -0.19 | 0.25 | 0.83 | 0.50-1.37 | 4.76 × 10^-1^ |
| Phylum *Actinobacteria* | Allergic asthma | IVW | 15 | -0.22 | 0.11 | 0.80 | 0.65-0.99 | 4.30 × 10^-2^ |
|  |  | Maximum likelihood | 15 | -0.21 | 0.11 | 0.81 | 0.65-1.00 | 5.17 × 10^-2^ |
|  |  | MR Egger | 15 | -0.28 | 0.46 | 0.75 | 0.31-1.85 | 5.47 × 10^-1^ |
|  |  | Weighted median | 15 | -0.26 | 0.14 | 0.77 | 0.58-1.02 | 6.82 × 10^-2^ |
|  |  | Weighted mode | 15 | -0.39 | 0.22 | 0.67 | 0.44-1.04 | 9.47 × 10^-2^ |
| Class *Deltaproteobacteria* | Eosinophilic asthma | IVW | 13 | -0.74 | 0.21 | 0.48 | 0.31-0.73 | **5.40 × 10^-4^** |
|  |  | Maximum likelihood | 13 | -0.74 | 0.22 | 0.48 | 0.31-0.73 | 6.79 × 10^-4^ |
|  |  | MR Egger | 13 | -0.72 | 0.59 | 0.49 | 0.15-1.55 | 2.51 × 10^-1^ |
|  |  | Weighted median | 13 | -0.78 | 0.30 | 0.46 | 0.26-0.82 | 8.22 × 10^-3^ |
|  |  | Weighted mode | 13 | -0.87 | 0.39 | 0.42 | 0.20-0.90 | 4.47 × 10^-2^ |
| Family *Desulfovibrionaceae* | Eosinophilic asthma | IVW | 10 | -0.55 | 0.22 | 0.58 | 0.37-0.89 | 1.34 × 10^-2^ |
|  |  | Maximum likelihood | 10 | -0.57 | 0.23 | 0.57 | 0.36-0.89 | 1.36 × 10^-2^ |
|  |  | MR Egger | 10 | -0.77 | 0.56 | 0.46 | 0.15-1.39 | 2.06 × 10^-1^ |
|  |  | Weighted median | 10 | -0.70 | 0.33 | 0.50 | 0.26-0.95 | 3.49 × 10^-2^ |
|  |  | Weighted mode | 10 | -0.73 | 0.41 | 0.48 | 0.22-1.08 | 1.11 × 10^-1^ |
| Genus *Sutterella* | Eosinophilic asthma | IVW | 12 | -0.45 | 0.21 | 0.63 | 0.42-0.96 | 3.01 × 10^-2^ |
|  |  | Maximum likelihood | 12 | -0.46 | 0.21 | 0.63 | 0.41-0.96 | 3.08 × 10^-2^ |
|  |  | MR Egger | 12 | -0.98 | 0.91 | 0.38 | 0.06-2.23 | 3.07 × 10^-1^ |
|  |  | Weighted median | 12 | -0.59 | 0.26 | 0.56 | 0.33-0.93 | 2.67 × 10^-2^ |
|  |  | Weighted mode | 12 | -0.75 | 0.45 | 0.47 | 0.19-1.14 | 1.24 × 10^-1^ |
| Order *Desulfovibrionales* | Eosinophilic asthma | IVW | 12 | -0.63 | 0.21 | 0.53 | 0.35-0.80 | 2.58 × 10^-3^ |
|  |  | Maximum likelihood | 12 | -0.65 | 0.22 | 0.52 | 0.34-0.80 | 3.10 × 10^-3^ |
|  |  | MR Egger | 12 | -0.66 | 0.53 | 0.52 | 0.18-1.47 | 2.45 × 10^-1^ |
|  |  | Weighted median | 12 | -0.75 | 0.31 | 0.47 | 0.26-0.87 | 1.64 × 10^-2^ |
|  |  | Weighted mode | 12 | -0.82 | 0.37 | 0.44 | 0.22-0.91 | 4.74 × 10^-2^ |
| Phylum *Verrucomicrobia* | Eosinophilic asthma | IVW | 12 | -0.43 | 0.19 | 0.65 | 0.45-0.95 | 2.53 × 10^-2^ |
|  |  | Maximum likelihood | 12 | -0.42 | 0.20 | 0.66 | 0.45-0.96 | 3.07 × 10^-2^ |
|  |  | MR Egger | 12 | -0.69 | 0.51 | 0.50 | 0.19-1.35 | 2.00 × 10^-1^ |
|  |  | Weighted median | 12 | -0.42 | 0.26 | 0.66 | 0.39-1.09 | 1.05 × 10^-1^ |
|  |  | Weighted mode | 12 | -0.16 | 0.44 | 0.85 | 0.36-2.04 | 7.27 × 10^-1^ |

MR, Mendelian randomization; SNP, single nucleotide polymorphism; Nsp, number of SNPs; OR, odds ratio; CI, confidence interval; IVW, inverse variance weighted.

Table S4. Causal associations between asthma and gut microbiota by using other four methods.

| **Exposure** | **Outcome** | **Method** | **Beta** | **SE** | **OR** | **95%CI** | ***P*** |
| --- | --- | --- | --- | --- | --- | --- | --- |
| Asthma | Family *Family_XIII* | Maximum likelihood | -0.05 | 0.02 | 0.95 | 0.91-1.00 | 2.91 × 10^-2^ |
|  |  | MR Egger | -0.10 | 0.07 | 0.90 | 0.79-1.03 | 1.38 × 10^-1^ |
|  |  | Weighted median | -0.06 | 0.03 | 0.94 | 0.89-1.01 | 7.35 × 10^-2^ |
|  |  | Weighted mode | -0.05 | 0.06 | 0.95 | 0.85-1.07 | 4.09 × 10^-1^ |
|  | Genus *Anaerofilum* | Maximum likelihood | 0.10 | 0.04 | 1.10 | 1.02-1.19 | 1.58 × 10^-2^ |
|  |  | MR Egger | 0.17 | 0.13 | 1.18 | 0.93-1.51 | 1.82 × 10^-1^ |
|  |  | Weighted median | 0.12 | 0.06 | 1.13 | 1.01-1.26 | 3.68 × 10^-2^ |
|  |  | Weighted mode | 0.17 | 0.10 | 1.18 | 0.97-1.45 | 1.11 × 10^-1^ |
|  | Genus *Anaerostipes* | Maximum likelihood | -0.05 | 0.02 | 0.95 | 0.91-1.00 | 3.62 × 10^-2^ |
|  |  | MR Egger | -0.01 | 0.07 | 0.99 | 0.87-1.13 | 8.93 × 10^-1^ |
|  |  | Weighted median | -0.06 | 0.03 | 0.94 | 0.89-1.00 | 5.86 × 10^-2^ |
|  |  | Weighted mode | -0.07 | 0.06 | 0.93 | 0.84-1.04 | 2.22 × 10^-1^ |
|  | Genus *Family_XIII_UCG001* | Maximum likelihood | -0.07 | 0.03 | 0.93 | 0.89-0.98 | 6.57 × 10^-3^ |
|  |  | MR Egger | -0.13 | 0.08 | 0.87 | 0.75-1.02 | 8.86 × 10^-2^ |
|  |  | Weighted median | -0.05 | 0.04 | 0.96 | 0.89-1.02 | 2.02 × 10^-1^ |
|  |  | Weighted mode | -0.02 | 0.07 | 0.98 | 0.85-1.13 | 8.02 × 10^-1^ |
|  | Genus *Lachnospiraceae_NK4A136_group* | Maximum likelihood | -0.04 | 0.02 | 0.96 | 0.92-1.00 | 4.06 × 10^-2^ |
|  |  | MR Egger | -0.06 | 0.07 | 0.94 | 0.83-1.07 | 3.62 × 10^-1^ |
|  |  | Weighted median | -0.04 | 0.03 | 0.96 | 0.90-1.02 | 2.08 × 10^-1^ |
|  |  | Weighted mode | -0.05 | 0.05 | 0.95 | 0.86-1.06 | 3.93 × 10^-1^ |
|  | Genus *Lachnospiraceae_UCG004* | Maximum likelihood | 0.05 | 0.02 | 1.06 | 1.01-1.11 | 2.36 × 10^-2^ |
|  |  | MR Egger | 0.13 | 0.08 | 1.13 | 0.98-1.32 | 1.04 × 10^-1^ |
|  |  | Weighted median | 0.06 | 0.04 | 1.06 | 0.99-1.14 | 8.75 × 10^-2^ |
|  |  | Weighted mode | 0.05 | 0.08 | 1.06 | 0.91-1.22 | 4.69 × 10^-1^ |
|  | Genus *Lachnospira* | Maximum likelihood | 0.29 | 0.09 | 1.34 | 1.12-1.60 | 1.16 × 10^-3^ |
|  |  | MR Egger | 0.11 | 0.47 | 1.12 | 0.45-2.80 | 8.24 × 10^-1^ |
|  |  | Weighted median | 0.38 | 0.12 | 1.47 | 1.17-1.84 | 1.03 × 10^-3^ |
|  |  | Weighted mode | 0.43 | 0.14 | 1.53 | 1.16-2.02 | 3.98 × 10^-2^ |
|  | Genus *Marvinbryantia* | Maximum likelihood | -0.08 | 0.03 | 0.93 | 0.88-0.98 | 4.05 × 10^-3^ |
|  |  | MR Egger | -0.22 | 0.08 | 0.81 | 0.69-0.94 | 8.69 × 10^-3^ |
|  |  | Weighted median | -0.08 | 0.04 | 0.92 | 0.85-1.00 | 4.17 × 10^-2^ |
|  |  | Weighted mode | -0.09 | 0.08 | 0.92 | 0.79-1.07 | 2.83 × 10^-1^ |
|  | Genus *Ruminococcus_torques_group* | Maximum likelihood | -0.05 | 0.02 | 0.95 | 0.91-0.99 | 2.47 × 10^-2^ |
|  |  | MR Egger | -0.13 | 0.07 | 0.88 | 0.77-1.01 | 7.13 × 10^-2^ |
|  |  | Weighted median | -0.06 | 0.03 | 0.94 | 0.89-1.00 | 4.14 × 10^-2^ |
|  |  | Weighted mode | -0.09 | 0.06 | 0.91 | 0.82-1.02 | 1.12 × 10^-1^ |

MR, Mendelian randomization; OR, odds ratio; CI, confidence interval.

Table S5. Full result of MR estimates for causal associations between four phenotypes of asthma and gut microbiota.

| **Exposure** | **Outcome** | **Method** | **Nsnp** | | **Beta** | **SE** | **OR** | **95%CI** | ***P*** |
| --- | --- | --- | --- | --- | --- | --- | --- | --- | --- |
|  | Genus *Parasutterella* | IVW | 22 | -0.06 | | 0.03 | 0.96 | 0.89-1.00 | 3.77 × 10^-2^ |
| Obesity related asthma |  | Maximum likelihood | 22 | -0.06 | | 0.03 | 0.94 | 0.89-0.99 | 2.38 × 10^-2^ |
|  |  | MR Egger | 22 | -0.24 | | 0.17 | 0.79 | 0.57-1.10 | 1.72 × 10^-1^ |
|  |  | Weighted median | 22 | -0.08 | | 0.04 | 0.93 | 0.86-1.00 | 3.88 × 10^-2^ |
|  |  | Weighted mode | 22 | -0.08 | | 0.07 | 0.92 | 0.81-1.05 | 2.36 × 10^-1^ |
|  | Genus *Ruminiclostridium6* | IVW | 22 | -0.05 | | 0.02 | 1.05 | 0.91-1.00 | 2.92 × 10^-2^ |
|  |  | Maximum likelihood | 22 | -0.05 | | 0.02 | 0.95 | 0.91-1.00 | 4.58 × 10^-2^ |
|  |  | MR Egger | 22 | -0.10 | | 0.13 | 0.90 | 0.69-1.17 | 4.47 × 10^-1^ |
|  |  | Weighted median | 22 | -0.06 | | 0.03 | 0.94 | 0.88-1.00 | 4.88 × 10^-2^ |
|  |  | Weighted mode | 22 | -0.06 | | 0.05 | 0.94 | 0.85-1.05 | 2.95 × 10^-1^ |
|  | Order *Bacillales* | IVW | 19 | 0.12 | | 0.05 | 1.32 | 1.02-1.24 | 2.20 × 10^-2^ |
|  |  | Maximum likelihood | 19 | 0.12 | | 0.05 | 1.13 | 1.02-1.25 | 1.96 × 10^-2^ |
|  |  | MR Egger | 19 | 0.29 | | 0.34 | 1.33 | 0.69-2.57 | 4.07 × 10^-1^ |
|  |  | Weighted median | 19 | 0.09 | | 0.07 | 1.09 | 0.95-1.26 | 2.30 × 10^-1^ |
|  |  | Weighted mode | 19 | 0.12 | | 0.14 | 1.13 | 0.85-1.48 | 4.12 × 10^-1^ |
|  | Class *Melainabacteria* | IVW | 16 | 0.09 | | 0.05 | 0.93 | 1.01-1.20 | 4.13 × 10^-3^ |
| Non-allergic asthma |  | Maximum likelihood | 16 | 0.10 | | 0.04 | 1.10 | 1.02-1.19 | 1.28 × 10^-2^ |
|  |  | MR Egger | 16 | 0.33 | | 0.18 | 1.39 | 0.99-1.97 | 8.07 × 10^-2^ |
|  |  | Weighted median | 16 | 0.06 | | 0.05 | 1.06 | 0.95-1.18 | 2.85 × 10^-1^ |
|  |  | Weighted mode | 16 | 0.05 | | 0.10 | 1.05 | 0.87-1.26 | 6.42 × 10^-1^ |
|  | Family *Enterobacteriaceae* | IVW | 16 | -0.07 | | 0.03 | 0.95 | 0.88-1.00 | 2.77 × 10^-2^ |
|  |  | Maximum likelihood | 16 | -0.07 | | 0.03 | 0.93 | 0.89-0.98 | 9.96 × 10^-3^ |
|  |  | MR Egger | 16 | -0.13 | | 0.14 | 0.88 | 0.67-1.15 | 3.55 × 10^-1^ |
|  |  | Weighted median | 16 | -0.04 | | 0.04 | 0.96 | 0.90-1.04 | 3.21 × 10^-1^ |
|  |  | Weighted mode | 16 | -0.02 | | 0.06 | 0.98 | 0.87-1.10 | 7.34 × 10^-1^ |
|  | Genus *Allisonella* | IVW | 13 | -0.13 | | 0.06 | 0.96 | 0.79-0.98 | 4.43 × 10^-2^ |
|  |  | Maximum likelihood | 13 | -0.13 | | 0.06 | 0.88 | 0.78-0.98 | 2.28 × 10^-2^ |
|  |  | MR Egger | 13 | -0.21 | | 0.26 | 0.81 | 0.48-1.36 | 4.40 × 10^-1^ |
|  |  | Weighted median | 13 | -0.07 | | 0.08 | 0.93 | 0.80-1.09 | 3.61 × 10^-1^ |
|  |  | Weighted mode | 13 | -0.02 | | 0.13 | 0.98 | 0.76-1.25 | 8.51 × 10^-1^ |
|  | Order *Enterobacteriales* | IVW | 16 | -0.07 | | 0.03 | 0.97 | 0.88-1.00 | 4.18 × 10^-2^ |
|  |  | Maximum likelihood | 16 | -0.07 | | 0.03 | 0.93 | 0.89-0.98 | 9.96 × 10^-3^ |
|  |  | MR Egger | 16 | -0.13 | | 0.14 | 0.88 | 0.67-1.15 | 3.55 × 10^-1^ |
|  |  | Weighted median | 16 | -0.04 | | 0.04 | 0.96 | 0.90-1.04 | 3.14 × 10^-1^ |
|  |  | Weighted mode | 16 | -0.02 | | 0.06 | 0.98 | 0.87-1.1 | 7.16 × 10^-1^ |
|  | Order *Gastranaerophilales* | IVW | 16 | 0.09 | | 0.05 | 0.97 | 1.01-1.20 | 4.06 × 10^-2^ |
|  |  | Maximum likelihood | 16 | 0.10 | | 0.04 | 1.10 | 1.02-1.19 | 1.39 × 10^-2^ |
|  |  | MR Egger | 16 | 0.33 | | 0.18 | 1.40 | 0.99-1.97 | 7.95 × 10^-2^ |
|  |  | Weighted median | 16 | 0.06 | | 0.05 | 1.06 | 0.95-1.18 | 2.75 × 10^-1^ |
|  |  | Weighted mode | 16 | 0.05 | | 0.09 | 1.05 | 0.88-1.25 | 5.94 × 10^-1^ |
|  | Phylum *Actinobacteria* | IVW | 16 | -0.04 | | 0.02 | 0.96 | 0.92-1.00 | 2.23 × 10^-2^ |
|  |  | Maximum likelihood | 16 | -0.05 | | 0.02 | 0.96 | 0.91-1.00 | 4.31 × 10^-2^ |
|  |  | MR Egger | 16 | -0.13 | | 0.09 | 0.88 | 0.74-1.05 | 1.68 × 10^-1^ |
|  |  | Weighted median | 16 | -0.05 | | 0.03 | 0.95 | 0.90-1.01 | 9.69 × 10^-2^ |
|  |  | Weighted mode | 16 | -0.06 | | 0.05 | 0.95 | 0.86-1.04 | 2.82 × 10^-1^ |
|  | Genus *Eubacterium_oxidoreducens_group* | IVW | 23 | 0.08 | | 0.04 | 0.94 | 1.01-1.00 | 4.61 × 10^-2^ |
| Allergic asthma |  | Maximum likelihood | 23 | 0.09 | | 0.04 | 1.09 | 1.01-1.18 | 2.63 × 10^-2^ |
|  |  | MR Egger | 23 | 0.21 | | 0.23 | 1.23 | 0.79-1.93 | 3.73 × 10^-1^ |
|  |  | Weighted median | 23 | 0.05 | | 0.05 | 1.05 | 0.95-1.17 | 3.20 × 10^-1^ |
|  |  | Weighted mode | 23 | 0.05 | | 0.10 | 1.06 | 0.86-1.29 | 6.06 × 10^-1^ |
|  | Genus *Ruminiclostridium6* | IVW | 23 | -0.06 | | 0.02 | 0.95 | 0.90-0.99 | 3.85 × 10^-2^ |
|  |  | Maximum likelihood | 23 | -0.06 | | 0.02 | 0.94 | 0.90-0.99 | 1.53 × 10^-2^ |
|  |  | MR Egger | 23 | -0.14 | | 0.14 | 0.87 | 0.66-1.14 | 3.25 × 10^-1^ |
|  |  | Weighted median | 23 | -0.04 | | 0.03 | 0.96 | 0.90-1.03 | 2.51 × 10^-1^ |
|  |  | Weighted mode | 23 | -0.01 | | 0.06 | 0.99 | 0.87-1.12 | 8.27 × 10^-1^ |
|  | Family *Rikenellaceae* | IVW | 19 | 0.03 | | 0.01 | 1.13 | 1.01-1.05 | 1.93 × 10^-2^ |
| Eosinophilic asthma |  | Maximum likelihood | 19 | 0.03 | | 0.01 | 1.03 | 1.01-1.05 | 6.88 × 10^-3^ |
|  |  | MR Egger | 19 | 0.06 | | 0.05 | 1.07 | 0.97-1.18 | 2.23 × 10^-1^ |
|  |  | Weighted median | 19 | 0.03 | | 0.02 | 1.03 | 1.001.06 | 5.89 × 10^-2^ |
|  |  | Weighted mode | 19 | 0.03 | | 0.02 | 1.03 | 0.98-1.08 | 2.34 × 10^-1^ |
|  | Genus *Alistipes* | IVW | 19 | 0.04 | | 0.01 | 1.10 | 1.01-1.06 | 3.67 × 10^-2^ |
|  |  | Maximum likelihood | 19 | 0.04 | | 0.01 | 1.04 | 1.01-1.06 | 1.29 × 10^-3^ |
|  |  | MR Egger | 19 | 0.05 | | 0.06 | 1.05 | 0.94-1.17 | 4.31 × 10^-1^ |
|  |  | Weighted median | 19 | 0.03 | | 0.02 | 1.03 | 1.00-1.07 | 4.98 × 10^-2^ |
|  |  | Weighted mode | 19 | 0.03 | | 0.02 | 1.03 | 0.99-1.08 | 1.83 × 10^-1^ |
|  | Genus *Catenibacterium* | IVW | 14 | 0.08 | | 0.03 | 0.94 | 1.02-1.15 | 4.33 × 10^-2^ |
|  |  | Maximum likelihood | 14 | 0.09 | | 0.03 | 1.09 | 1.02-1.16 | 6.22 × 10^-3^ |
|  |  | MR Egger | 14 | 0.28 | | 0.19 | 1.33 | 0.91-1.93 | 1.70 × 10^-1^ |
|  |  | Weighted median | 14 | 0.09 | | 0.04 | 1.09 | 1.01-1.18 | 3.65 × 10^-2^ |
|  |  | Weighted mode | 14 | 0.09 | | 0.07 | 1.10 | 0.96-1.25 | 2.09 × 10^-1^ |
|  | Genus *Eubacterium_fissicatena_group* | IVW | 17 | 0.07 | | 0.03 | 0.88 | 1.02-1.13 | 2.31 × 10^-2^ |
|  |  | Maximum likelihood | 17 | 0.07 | | 0.03 | 1.07 | 1.02-1.13 | 5.91 × 10^-3^ |
|  |  | MR Egger | 17 | 0.28 | | 0.14 | 1.33 | 1.01-1.74 | 5.74 × 10^-2^ |
|  |  | Weighted median | 17 | 0.10 | | 0.04 | 1.11 | 1.03-1.19 | 4.59 × 10^-3^ |
|  |  | Weighted mode | 17 | 0.13 | | 0.06 | 1.13 | 1.00-1.29 | 7.04 × 10^-2^ |
|  | Genus *Flavonifractor* | IVW | 19 | -0.03 | | 0.02 | 0.94 | 0.94-1.00 | 4.33 × 10^-2^ |
|  |  | Maximum likelihood | 19 | -0.03 | | 0.01 | 0.97 | 0.94-1.00 | 3.52 × 10^-2^ |
|  |  | MR Egger | 19 | -0.03 | | 0.07 | 0.97 | 0.85-1.11 | 7.04 × 10^-1^ |
|  |  | Weighted median | 19 | -0.03 | | 0.02 | 0.97 | 0.93-1.01 | 1.24 × 10^-1^ |
|  |  | Weighted mode | 19 | -0.04 | | 0.03 | 0.96 | 0.90-1.02 | 2.14 × 10^-1^ |
|  | Genus *Hungatella* | IVW | 17 | 0.05 | | 0.02 | 1.10 | 1.01-1.11 | 3.69 × 10^-2^ |
|  |  | Maximum likelihood | 17 | 0.06 | | 0.02 | 1.06 | 1.01-1.11 | 2.36 × 10^-2^ |
|  |  | MR Egger | 17 | 0.10 | | 0.13 | 1.10 | 0.85-1.43 | 4.83 × 10^-1^ |
|  |  | Weighted median | 17 | 0.05 | | 0.03 | 1.05 | 0.98-1.12 | 1.68 × 10^-1^ |
|  |  | Weighted mode | 17 | 0.05 | | 0.05 | 1.05 | 0.95-1.15 | 3.62 × 10^-1^ |
|  | Genus *Methanobrevibacter* | IVW | 17 | 0.05 | | 0.03 | 0.96 | 1.00-1.11 | 4.24 × 10^-2^ |
|  |  | Maximum likelihood | 17 | 0.05 | | 0.03 | 1.05 | 1.00-1.11 | 4.75 × 10^-2^ |
|  |  | MR Egger | 17 | 0.21 | | 0.14 | 1.24 | 0.94-1.64 | 1.55 × 10^-1^ |
|  |  | Weighted median | 17 | 0.04 | | 0.04 | 1.05 | 0.97-1.12 | 2.22 × 10^-1^ |
|  |  | Weighted mode | 17 | 0.04 | | 0.04 | 1.05 | 0.96-1.14 | 3.31 × 10^-1^ |

MR, Mendelian randomization; SNP, single nucleotide polymorphism; Nsp, number of SNPs; OR, odds ratio; CI, confidence interval; IVW, inverse variance weighted.

Table S8. The sensitivity analyses of causality between gut microbiota and asthma and its phenotypes based on MR results.

| **Exposure** | | **Outcome** | **Horizontal pleiotropy** | | | **Heterogeneity** | | | |
| --- | --- | --- | --- | --- | --- | --- | --- | --- | --- |
|  | |  |  |  |  | IVW | | MR Egger | |
|  | |  | Egger intercept | SE | *P* | Cochran’s Q | *P* | Cochran’s Q | *P* |
| Class *Actinobacteria* | | Asthma | -5.40 × 10^-3^ | 1.10 × 10^-2^ | 0.63 | 14.39 | 0.42 | 14.13 | 0.37 |
| Class *Bacilli* | |  | -8.10 × 10^-3^ | 1.10 × 10^-2^ | 0.48 | 18.89 | 0.33 | 18.30 | 0.31 |
| Family *Pasteurellaceae* | |  | -9.20 × 10^-3^ | 1.00 × 10^-2^ | 0.39 | 9.86 | 0.71 | 9.05 | 0.70 |
| Genus *Lachnospiraceae_UCG001* | |  | -1.70 × 10^-2^ | 2.20 × 10^-2^ | 0.45 | 17.01 | 0.15 | 16.12 | 0.14 |
| Genus *Oxalobacter* | |  | 1.90 × 10^-3^ | 2.40 × 10^-2^ | 0.94 | 7.71 | 0.66 | 7.71 | 0.56 |
| Genus *Ruminococcus* | |  | 5.30 × 10^-4^ | 1.00 × 10^-2^ | 0.96 | 14.95 | 0.38 | 0.66 | 0.31 |
| Order *Lactobacillales* | |  | -9.50 × 10^-4^ | 1.10 × 10^-2^ | 0.94 | 14.33 | 0.43 | 14.32 | 0.35 |
| Order *NB1n* | |  | 4.00 × 10^-3^ | 1.80 × 10^-2^ | 0.83 | 12.44 | 0.41 | 12.38 | 0.34 |
| Order *Pasteurellales* | |  | -9.20 × 10^-3^ | 1.00 × 10^-2^ | 0.39 | 9.86 | 0.71 | 9.05 | 0.70 |
| Class *Deltaproteobacteria* | | Obesity related asthma | -2.10 × 10^-3^ | 2.20 × 10^-2^ | 0.92 | 11.29 | 0.50 | 11.28 | 0.42 |
| Family *Family_XIII* | |  | -1.10 × 10^-2^ | 4.00 × 10^-2^ | 0.78 | 7.90 | 0.44 | 7.81 | 0.35 |
| Family *Pasteurellaceae* | |  | -1.40 × 10^-2^ | 2.30 × 10^-2^ | 0.55 | 16.23 | 0.24 | 15.74 | 0.20 |
| Family *Rikenellaceae* | |  | 2.00 × 10^-2^ | 2.40 × 10^-2^ | 0.43 | 10.70 | 0.83 | 10.03 | 0.82 |
| Genus *Anaerofilum* | |  | 5.60 × 10^-2^ | 4.60 × 10^-2^ | 0.26 | 8.99 | 0.53 | 7.54 | 0.58 |
| Genus *Collinsella* | |  | -1.50 × 10^-3^ | 3.90 × 10^-2^ | 0.97 | 5.36 | 0.72 | 5.35 | 0.62 |
| Genus *Ruminococcaceae_UCG010* | |  | 1.20 × 10^-2^ | 3.50 × 10^-2^ | 0.75 | 6.02 | 0.30 | 5.84 | 0.21 |
| Genus *Terrisporobacter* | |  | -4.10 × 10^-2^ | 4.00 × 10^-2^ | 0.38 | 2.63 | 0.62 | 1.56 | 0.67 |
| Genus *Turicibacter* | |  | 8.00 × 10^-2^ | 4.60 × 10^-2^ | 0.12 | 7.68 | 0.57 | 4.63 | 0.80 |
| Order *Bacillales* | |  | 1.50 × 10^-2^ | 5.10 × 10^-2^ | 0.77 | 10.32 | 0.24 | 10.19 | 0.18 |
| Order *NB1n* | |  | 6.70 × 10^-3^ | 3.30 × 10^-2^ | 0.84 | 6.64 | 0.83 | 6.64 | 0.83 |
| Order *Pasteurellales* | |  | -1.40 × 10^-2^ | 2.30 × 10^-2^ | 0.55 | 16.23 | 0.24 | 15.74 | 0.20 |
| Class *Bacilli* | |  | 2.80 × 10^-2^ | 2.20 × 10^-2^ | 0.24 | 18.26 | 0.37 | 16.69 | 0.41 |
| Family *Acidaminococcaceae* |  | | 6.90 × 10^-2^ | 4.20 × 10^-2^ | 0.16 | 8.50 | 0.20 | 5.50 | 0.36 |
| Genus *Desulfovibrio* | |  | 2.70 × 10^-2^ | 3.40 × 10^-2^ | 0.45 | 7.00 | 0.64 | 6.38 | 0.60 |
| Genus *Eubacterium_Nodatum_Group* | | Non-allergic asthma | -2.80 × 10^-3^ | 4.20 × 10^-2^ | 0.95 | 4.82 | 0.90 | 4.81 | 0.85 |
| Genus *Eubacterium_Xylanophilum_Group* | |  | 1.40 × 10^-2^ | 3.10 × 10^-2^ | 0.67 | 5.27 | 0.73 | 5.08 | 0.65 |
| Genus *Oxalobacter* | |  | -4.20 × 10^-3^ | 5.10 × 10^-2^ | 0.94 | 9.50 | 0.49 | 9.49 | 0.39 |
| Class *Bacteroidia* | | Allergic asthma | 2.30 × 10^-2^ | 1.70 × 10^-2^ | 0.20 | 8.61 | 0.80 | 6.74 | 0.87 |
| Genus *Ruminococcaceae_UCG009* | |  | -3.60 × 10^-2^ | 3.10 × 10^-2^ | 0.27 | 8.61 | 0.80 | 6.74 | 0.87 |
| Order *Bacteroidales* | |  | 2.30 × 10^-2^ | 1.70 × 10^-2^ | 0.20 | 8.61 | 0.80 | 6.74 | 0.87 |
| Phylum *Actinobacteria* | |  | 4.00 × 10^-3^ | 2.80 × 10^-2^ | 0.89 | 5.66 | 0.97 | 5.64 | 0.96 |
| Class *Deltaproteobacteria* | | Eosinophilic asthma | -2.10 × 10^-3^ | 4.30 × 10^-2^ | 0.96 | 13.08 | 0.36 | 13.07 | 0.29 |
| Family *Desulfovibrionaceae* | |  | 1.90 × 10^-2^ | 4.30 × 10^-2^ | 0.68 | 8.86 | 0.45 | 8.67 | 0.37 |
| Genus *Sutterella* | |  | 3.60 × 10^-2^ | 6.10 × 10^-2^ | 0.57 | 4.40 | 0.96 | 4.05 | 0.95 |
| Order *Desulfovibrionales* | |  | 1.80 × 10^-3^ | 3.90 × 10^-2^ | 0.96 | 10.14 | 0.52 | 10.14 | 0.43 |
| Phylum *Verrucomicrobia* | |  | 2.50 × 10^-2^ | 4.50 × 10^-2^ | 0.59 | 8.46 | 0.67 | 8.15 | 0.61 |

MR, Mendelian randomization; SE, standard error; IVW, inverse variance weighted.

Table S9. The sensitivity analyses of causality between asthma and its phenotypes and gut microbiota based on MR results.

| **Exposure** | **Outcome** | **Horizontal pleiotropy** | | | **Heterogeneity** | | | |
| --- | --- | --- | --- | --- | --- | --- | --- | --- |
|  |  |  |  |  | IVW | | MR Egger | |
|  |  | Egger intercept | SE | *P* | Cochran’s Q | *P* | Cochran’s Q | *P* |
| Asthma | Family *Family_XIII* | 4.60 × 10^-3^ | 5.60 × 10^-3^ | 0.41 | 54.80 | 0.67 | 54.11 | 0.66 |
|  | Genus *Anaerofilum* | -6.30 × 10^-3^ | 1.00 × 10^-2^ | 0.54 | 53.20 | 0.65 | 52.82 | 0.63 |
|  | Genus *Anaerostipes* | -3.30 × 10^-3^ | 5.50 × 10^-3^ | 0.56 | 59.09 | 0.51 | 58.74 | 0.48 |
|  | Genus *Family_XIII_UCG001* | 5.60 × 10^-3^ | 6.40 × 10^-3^ | 0.38 | 46.90 | 0.87 | 46.13 | 0.87 |
|  | Genus *Lachnospiraceae_NK4A136_Group* | 1.40 × 10^-3^ | 5.50 × 10^-3^ | 0.80 | 51.21 | 0.78 | 51.14 | 0.76 |
|  | Genus *Lachnospiraceae_UCG004* | -6.30 × 10^-3^ | 6.20 × 10^-3^ | 0.32 | 63.35 | 0.33 | 62.27 | 0.33 |
|  | Genus *Lachnospira* | 1.30 × 10^-2^ | 3.50 × 10^-2^ | 0.74 | 8.24 | 0.08 | 7.90 | 0.05 |
|  | Genus *Marvinbryantia* | 1.20 × 10^-2^ | 6.60 × 10^-3^ | 0.07 | 61.87 | 0.37 | 58.37 | 0.46 |
|  | Genus *Ruminococcus_Torques_Group* | 6.70 × 10^-3^ | 5.60× 10^-3^ | 0.24 | 65.33 | 0.30 | 63.82 | 0.31 |
| Obesity related asthma | genus *Parasutterella* | 5.50 × 10^-2^ | 5.00 × 10^-2^ | 0.30 | 28.68 | 0.12 | 27.11 | 0.13 |
|  | genus *Ruminiclostridium6* | 9.80 × 10^-2^ | 3.50 × 10^-2^ | 0.02 | 18.72 | 0.60 | 18.55 | 0.55 |
|  | order *Bacillales* | 9.50 × 10^-3^ | 6.40 × 10^-2^ | 0.89 | 15.70 | 0.61 | 15.45 | 0.56 |
| Non-allergic asthma | class *Melainabacteria* | -5.20 × 10^-2^ | 4.10 × 10^-2^ | 0.23 | 21.12 | 0.13 | 18.57 | 0.18 |
|  | family *Enterobacteriaceae* | 6.20 × 10^-2^ | 3.20 × 10^-2^ | 0.08 | 25.12 | 0.05 | 24.69 | 0.04 |
|  | genus *Allisonella* | 5.80 × 10^-2^ | 3.70 × 10^-2^ | 0.14 | 6.74 | 0.87 | 6.64 | 0.83 |
|  | order *Enterobacteriales* | -3.90 × 10^-2^ | 3.80 × 10^-2^ | 0.33 | 25.12 | 0.05 | 24.69 | 0.04 |
|  | order *Gastranaerophilales* | -2.10 × 10^-3^ | 2.20 × 10^-2^ | 0.92 | 21.23 | 0.13 | 18.63 | 0.18 |
|  | phylum *Actinobacteria* | -1.10 × 10^-2^ | 4.00 × 10^-2^ | 0.78 | 7.90 | 0.93 | 6.92 | 0.94 |
| Allergic asthma | genus *Eubacterium_oxidoreducens_group* | -1.40 × 10^-2^ | 2.30 × 10^-2^ | 0.55 | 23.28 | 0.39 | 22.94 | 0.35 |
|  | genus *Ruminiclostridium6* | 2.00 × 10^-2^ | 2.40 × 10^-2^ | 0.43 | 22.56 | 0.43 | 22.17 | 0.39 |
| Eosinophilic asthma | family *Rikenellaceae* | 5.60 × 10^-2^ | 4.60 × 10^-2^ | 0.26 | 18.58 | 0.42 | 18.10 | 0.38 |
|  | genus *Alistipes* | -1.50 × 10^-3^ | 3.90 × 10^-2^ | 0.97 | 24.13 | 0.15 | 24.09 | 0.12 |
|  | genus *Catenibacterium* | 1.20 × 10^-2^ | 3.50 × 10^-2^ | 0.75 | 11.90 | 0.54 | 10.81 | 0.55 |
|  | genus *Eubacterium_fissicatena_group* | -4.10 × 10^-2^ | 4.00 × 10^-2^ | 0.38 | 13.91 | 0.61 | 11.43 | 0.72 |
|  | genus *Flavonifractor* | 8.00 × 10^-2^ | 4.60 × 10^-2^ | 0.12 | 20.17 | 0.32 | 20.16 | 0.27 |
|  | genus *Hungatella* | 1.50 × 10^-2^ | 5.10 × 10^-2^ | 0.77 | 15.85 | 0.46 | 15.75 | 0.40 |
|  | genus *Methanobrevibacter* | 6.70 × 10^-3^ | 3.30 × 10^-2^ | 0.84 | 15.51 | 0.49 | 14.19 | 0.51 |

MR, Mendelian randomization; SE, standard error; IVW, inverse variance weighted.


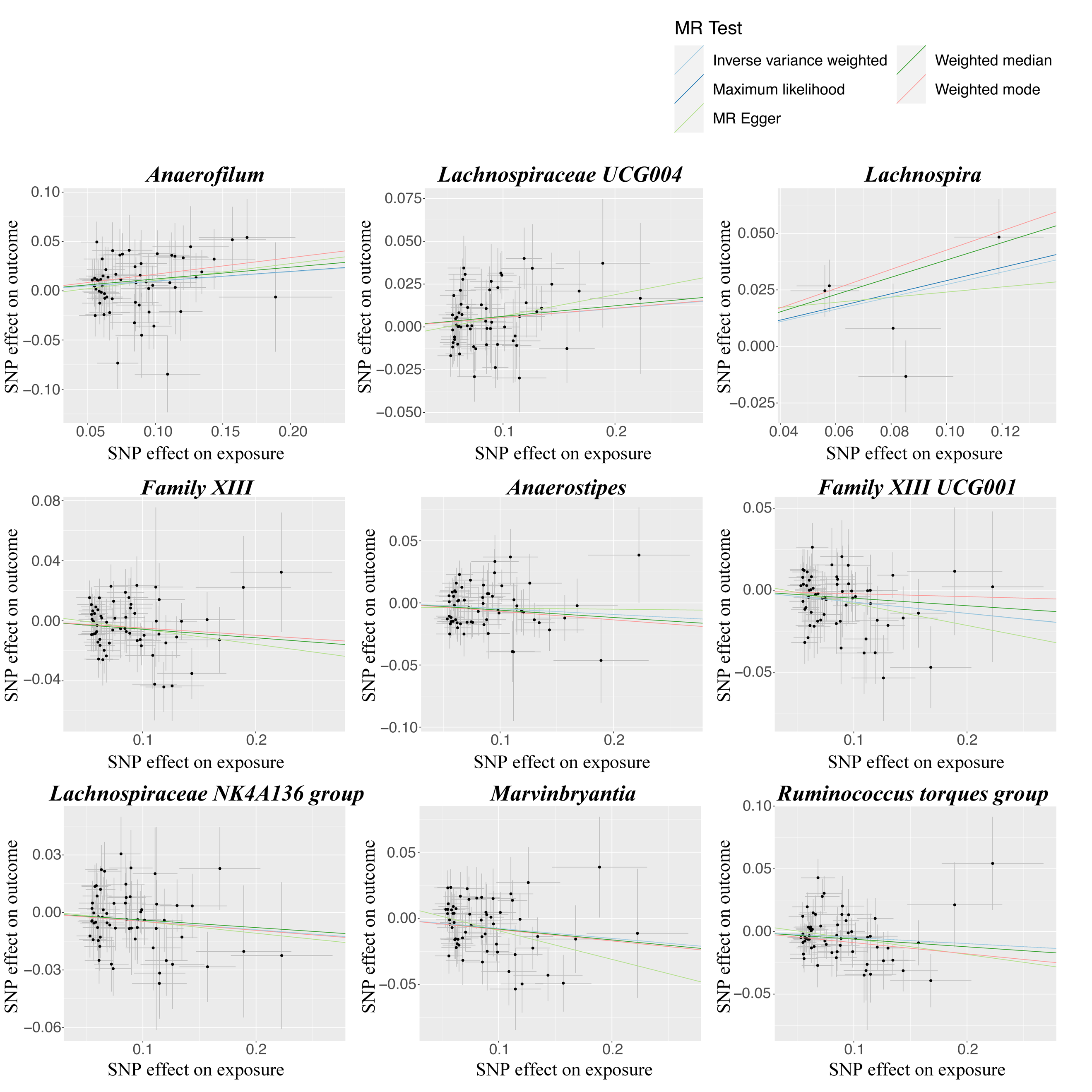


**Fig. S1** Scatter plots for the causal association between asthma and gut microbiota

**Fig. S2** Leave-one-out plots for the causal association between gut microbiota and asthma

**Fig. S3** Leave-one-out plots for the causal association between asthma and gut microbiota
